# A high-quality annotated transcriptome of swine peripheral blood

**DOI:** 10.1186/s12864-017-3863-7

**Published:** 2017-06-24

**Authors:** Haibo Liu, Timothy P.L. Smith, Dan J. Nonneman, Jack C.M. Dekkers, Christopher K. Tuggle

**Affiliations:** 1 Bioinformatics and Computational Biology Program, Department of Animal Science, Iowa State University, 2258 Kildee Hall, Ames, IA 50011 USA; 2 USDA, ARS, U.S. Meat Animal Research Center, Clay Center, NE 68933 USA; 3 Department of Animal Science, Iowa State University, 239 Kildee Hall, Ames, IA 50011 USA; 4 Department of Animal Science, Iowa State University, 2255 Kildee Hall, Ames, IA 50011 USA

**Keywords:** *Sus scrofa*, Peripheral blood, De novo transcriptome assembly, Genome-guided transcriptome assembly

## Abstract

**Background:**

High throughput gene expression profiling assays of peripheral blood are widely used in biomedicine, as well as in animal genetics and physiology research. Accurate, comprehensive, and precise interpretation of such high throughput assays relies on well-characterized reference genomes and/or transcriptomes. However, neither the reference genome nor the peripheral blood transcriptome of the pig have been sufficiently assembled and annotated to support such profiling assays in this emerging biomedical model organism. We aimed to assemble published and novel RNA-seq data to provide a comprehensive, well-annotated blood transcriptome for pigs by integrating a de novo assembly with a genome-guided assembly.

**Results:**

A de novo and a genome-guided transcriptome of porcine whole peripheral blood was assembled with ~162 million pairs of paired-end and ~183 million single-end, trimmed and normalized Illumina RNA-seq reads (~6 billion initial reads from 146 RNA-seq libraries) from five independent studies by using the Trinity and Cufflinks software, respectively. We then removed putative transcripts (PTs) of low confidence from both assemblies and merged the remaining PTs into an integrated transcriptome consisting of 132,928 PTs, with 126,225 (~95%) PTs from the de novo assembly and more than 91% of PTs spliced. In the integrated transcriptome, ~90% and 63% of PTs had significant sequence similarity to sequences in the NCBI NT and NR databases, respectively; 68,754 (~52%) PTs were annotated with 15,965 unique gene ontology (GO) terms; and 7618 PTs annotated with Enzyme Commission codes were assigned to 134 pathways curated by the Kyoto Encyclopedia of Genes and Genomes (KEGG). Full exon-intron junctions of 17,528 PTs were validated by PacBio IsoSeq full-length cDNA reads from 3 other porcine tissues, NCBI pig RefSeq mRNAs and transcripts from Ensembl *Sscrofa10.2* annotation. Completeness of the 5’ termini of 37,569 PTs was validated by public cap analysis of gene expression (CAGE) data. By comparison to the Ensembl transcripts, we found that (1) the deduced precursors of 54,402 PTs shared at least one intron or exon with those of 18,437 Ensembl transcripts; (2) 12,262 PTs had both longer 5’ and 3’ termini than their maximally overlapping Ensembl transcripts; and (3) 41,838 spliced PTs were totally missing from the *Sscrofa10.2* annotation. Similar results were obtained when the PTs were compared to the pig NCBI RefSeq mRNA collection.

**Conclusions:**

We built, validated and annotated a comprehensive porcine blood transcriptome with significant improvement over the annotation of Ensembl *Sscrofa10.2* and the pig NCBI RefSeq mRNAs, and laid a foundation for blood-based high throughput transcriptomic assays in pigs and for advancing annotation of the pig genome.

**Electronic supplementary material:**

The online version of this article (doi:10.1186/s12864-017-3863-7) contains supplementary material, which is available to authorized users.

## Background

In higher animals, peripheral blood is a complex and informative tissue type, consisting of acellular plasma and multiple types of cells at various differentiation states [1, 2]. Peripheral blood cells can be roughly classified into anucleated red blood cells (RBCs) and platelets, and nucleated white blood cells (WBCs), with WBCs most transcriptionally active [1, 3–8]. The WBCs can be further divided into neutrophils, eosinophils, basophils, B lymphocytes, T lymphocytes and monocytes. Each cell type is composed of continuously differentiating subtypes, leading to variability in RNA content of the overall peripheral blood transcriptome. Peripheral blood is also a highly dynamic tissue type with a high rate of cell turnover, resulting in high diversity of RNA content over time [9]. In addition, peripheral blood interacts with every organ and tissue in the body, and consequently presents gene expression profiles that can reflect the physiopathological status, behaviors, growth stage and lifestyle of subjects [2, 10–16]. Combined with easy and minimally invasive accessibility, the reflective relationship to body status makes peripheral blood a highly desirable tissue type for disease prediction, diagnosis, monitoring, prognosis, and biomarker development [2, 17].

Due to the high similarity in anatomy, genetics and physiology of pigs and humans, pigs have been recognized as a more appropriate animal model for many human diseases than rodents [18–20]. However, the widely used swine reference genome *Sscrofa10.2* (SSC10.2 for short) [20] is neither well-assembled nor well-annotated [21]. For example, only 43% of the 25,510 transcripts of the 21,630 coding genes have both a 5′ and 3′ UTRs defined. On average, less than 1.2 isoforms per protein-coding gene and only 3124 non-coding genes have been identified in SSC10.2. More recently, Warr reported that more than 33% of SSC10.2 is not correctly assembled or is otherwise unreliable [22]. In addition, there are many gaps and thousands of fragmented, unplaced contigs in the assembly, with many known genes missing from the reference genome [22, 23]. Furthermore, the approximate number of genes with detectable expression in porcine peripheral blood is still unknown. The lack of a detailed catalog and annotation of the pig genome and transcriptome hinders annotation-based high throughput studies [24, 25]. Fortunately, two independent assemblies of the pig genome have been recently initiated using a PacBio long read-based approach [22, 26] and are currently being annotated.

Public porcine RNA-seq data have rapidly accumulated over the past few years and are continuously being produced in labs around the world. These data are valuable resources for evidence-based genome annotation, due to their broad coverage, great depth and high resolution [27–29]. The unassembled RNA-seq reads can be used for genome annotation by mapping them directly to the reference genome, but an assembled transcriptome is more useful and less susceptible to mapping artifacts. In addition, RNA-seq read-based transcriptome assembly can reduce the amount of information to be tracked, help identify transcript boundaries and splice isoforms, and facilitate the discovery of novel transcripts whose genes are not included in the reference genome [29]. Currently, there are two popular strategies for transcriptome assembly: de novo assembly and genome-guided assembly, each with its own advantages and disadvantages [30, 31]. Integration of de novo assembly and genome-guided assembly, also known as a hybrid transcriptome assembly method, was proposed as an effective way to reconstruct comprehensive transcriptomes [30, 32, 33].

We here report the build and annotation of a comprehensive blood transcriptome by using blood RNA-seq data from several independent studies using pigs of different genetic backgrounds and physiopathological conditions via the hybrid transcriptome assembly strategy. The resulting integrated transcriptome was assessed, validated and extensively annotated with different sources of knowledge and evidence. This integrated transcriptome lays a reliable foundation for future blood-based high throughput gene expression studies of pig diseases and physiology, and will also contribute towards annotation of the emerging improved porcine reference genome assemblies.

## Methods

### Animal handling and RNA-seq data collection

Publicly available porcine peripheral blood Illumina RNA-seq data were downloaded from the European Nucleotide Archive (ENA) and Ensembl FTP sites, while RNA-seq data from lipopolysaccharide (LPS)- or saline-treated pigs were from an unpublished study in our lab (H. Liu, K. Feye et al., unpublished). A detailed description of these RNA-seq data is available in Table 1. In summary, over 6 billion Illumina RNA-seq reads from146 independent libraries made of blood RNA samples from 76 pigs of different breeds, age, treatments and health status were used for the blood transcriptome assembly [34–37].

**Table 1:** RNA-seq data used for the blood transcriptome assembly

Study accession	Sample description	Read length	Layout	Total raw read count	Breed	Source	Run accession	Reference
PRJEB5250	16 pigs (7 weeks old) were infected with *Salmonella enterica serovar Typhimurium*. Whole blood was sampled before infection (day 0) and day 2 post infection. Globin was depleted from the total RNA.	51	SE	1,611,707,563	Yorkshire crossbred	ENA	ERR413315 - ERR413346	[35]
PRJNA189967	Whole blood from 3 individual healthy pigs	51	SE	106,157,368	unknown	ENA	SRR747924-SRR747926	[36]
Swine Genome Sequencing Consortium	Whole blood from one healthy pig	83	SE	48,973,230	Duroc	ENSEMBL(ftp://ftp.ensembl.org/pub/release-83/bamcov/sus_scrofa/genebuild/whole_blood_merged_sorted.bam)	Unknown	http://www.ensembl.org/info/genome/genebuild/2012_04_sus_scrofa_genebuild.pdf
PRJEB12300	Whole blood was sampled from 31 post-weaning (5 ~ 6 weeks old) pigs from lines divergently selected for residual feed intake. Globin was depleted from the total RNA.	100	PE	2 × 632,557,790	Yorkshire	ENA	ERR1199492-ERR1199522	[34]
PRJEB20136	28 pigs (~ 63 kg of body weight) from lines divergently selected for residual feed intake were muscularly injected with *E. coli* LPS or saline at time 0, and 48, 96 h and 144 h post the first injection. Whole blood was sampled immediately before first injection and 2, 6, 24 and 168 h post first injection. Globin was depleted from the total RNA.	49	PE	2 × 1,509,173,972	Yorkshire	ENA	ERR1898446-ERR1898477	H. Liu, K. Feye et al., unpublished

The sources of pigs and how they were cared and handled in the published studies were as described [34–36]. The pigs treated with LPS or saline were from the generation 8 of two Yorkshire lines divergently selected for residual feed intake at Iowa State University [38, 39], and were euthanized by barbiturate overdose and exsanguinated by following an protocol approved by the Institutional Animal Care and Use Committee (IACUC) at Iowa State University.

### Pig reference genomes

The sequence and annotation file of the pig reference genome, SSC10.2, were downloaded from the Ensembl FTP site [40]. Another pig reference genome, USMARCv1.0, was recently assembled using 65× coverage of PacBio long reads from DNA of lung tissue of a cross-bred pig (½ Landrace- ¼ Duroc- ¼ Yorkshire) and is on the order of 100× more contiguous than SSC10.2 (T. Smith et al. unpublished).

The pig used for the USMARCv1.0 reference genome assembly was from a composite population created and maintained at the US Meat Animal Research Center (USMARC). The animal was euthanized by electrical stunning, exsanguinated and harvested using standard humane methods at the USMARC abattoir under the supervision of USDA-FSIS inspection. Care and handling of all animals used in this study was approved by the IACUC at USMARC under experimental outline 5438–31000–83-02.

### Preprocessing and normalizing Illumina RNA-seq reads

Quality of the raw RNA-seq data was first assessed with FASTQC (v0.10.1) [41] for each library. Then, the Illumina sequencing adaptor sequences and low quality bases were trimmed from the raw reads by using the Trimmomatic software (v0.32) [42] in a sliding window mode with options: *ILLUMINACLIP:adapters.fa:2:30:10:1:true LEADING:3 TRAILING:3 SLIDINGWINDOW:4:20 LEADING:3 TRAILING:3 MINLEN:25*, such that the average base quality score (Phred +33) was at least 20 for every 4-base sliding window, the quality scores of leading and trailing bases were at least 3 and the minimum length of kept reads was 25 bases. Because large amounts of reads were available for the transcriptome assembly, to reduce cost and runtime of de novo assembly, the trimmed reads were in silico normalized by using a *k*-mer abundance-based utility, *insilico_read_normalization.pl*, in the Trinity package (v2.1.1) [43, 44]. The paired-end and single-end reads were normalized separately with the following explicit option settings: *--max_cov 100 --pairs_together --PARALLEL_STATS* for paired-end reads and *--max_cov 100* for single-end reads.

### Blood transcriptome assembly

The normalized paired-end and single-end reads from peripheral blood were combined and assembled by using the de novo Trinity software (v2.1.1), with an explicit option setting *--min_kmer_cov 2*, as described in [43]. By default, only assembled products longer than 200 bases were output. To supplement the de novo transcriptome, a genome-guided transcriptome assembly was also performed as follows. First, the normalized paired-end and single-end reads were separately aligned to the USMARCv1.0 reference genome by using the STAR (v2.4.1a) software [45] with Cufflinks-compatible options. The alignment output was subsequently sorted and merged after format conversion. The resulting BAM file was used for genome-guided transcriptome assembly independent of genome annotation by using the Cufflinks software (v2.2.1) with the following option settings: *-F 0.10 -u -j 0.25 -I 1000000 --min-intron-length 20 --overlap-radius 10* [46]. Assembled products of 200 bases or shorter were discarded before further analysis. Hereafter, the assembled products from both the de novo and genome-guided assemblies were called putative transcripts (PTs).

### Quality assessment, filtering, and annotation of the transcriptome assemblies

The normalized RNA-seq reads were mapped back to the de novo transcriptome assembly and the mapping results were assessed using Trinity utilities, as described in [43, 44]. The number of full-length PTs in the de novo transcriptome was estimated based on the percentage of coverage of manually curated protein sequences and porcine RefSeq mRNA sequences in the Swiss-Prot and the NCBI NT databases by PTs as determined by using BLASTX and BLASTN software (v2.5.0) with E-value cutoffs of 10^−10^ and 10^−20^, respectively [43].

The coding potential of each PT was predicted by using the PLEK software (v1.2) based on the default classification model for human transcripts [47]. The PLEK software is an efficient long noncoding RNA (lncRNAs) predicting tool, which can distinguish lncRNAs from mRNAs based on an improved *k*-mer scheme and a support vector machine algorithm, independent of genomic sequences and annotation [47]. PTs with coding potentials less than zero were considered lncRNAs, with the remaining PTs considered as protein-coding PTs. The coverage per base (CPB) was calculated by using the Bedtools *genomecov* utility (v2.26.0) [48] based on the normalized reads mapped to the USMARCv1.0 genome assembly as above.

The genomic origins of the PTs in the de novo assembly were determined by mapping them to the two reference genomes, SSC10.2 and USMARCv1.0, using the GMAP software (version 2016-09-23) [49] with the following option settings: *-n 0 -f samse*. Spliced versus unspliced transcripts were determined based on the alignment results. A uniquely mapping PT was considered spliced if there was at least one “N” in its CIGAR (Concise Idiosyncratic Gapped Alignment Report) column of the SAM file; a translocation mapping transcript was considered spliced if it was mapped to two locations on two different scaffolds or more than 2 million bases apart on the same scaffold, and there was at least one “N” in its CIGAR column of the SAM file for either mapped part. The splice status of transcripts that mapped to multiple locations or that were not mappable was not determined. The splice status of PTs in the genome-guided assembly was determined based on the number of exons of each PT in the GTF (Gene Transfer Format) file output by Cufflinks.

PTs not derived from the pig genome were identified for removal from the de novo assembly by comparing their sequences to the NCBI NT database through running DC-megaBLAST. Only alignments with E-value  ≤ 10^−20^ were considered significant to insure that true pig transcripts were not inadvertently removed. A PT was considered “contaminant” if its top match was a non-vertebrate sequence, unless it had a better scored alignment with the pig reference genomes than to the non-vertebrate sequence. The PTs in the de novo assembly were also aligned to sequences in the NCBI NR database with BLASTX, using the following explicit option settings: *-outfmt 5 -evalue 1e-6 -word_size 5 -show_gis -num_alignments 10 -task blastx-fast -max_hsps 20*, which provided protein-level annotation of transcripts in the de novo assembly. Spliced PTs and unspliced non-intronic PTs in the genome-guided assembly were aligned with sequences in the NT and NR databases by using DC-megaBLAST and BLASTX with the same option settings as above.

One possible type of “transcriptional noise” is unspliced PTs that overlap intron intervals, either with or without exonic sequence (collectively called unspliced intronic PTs). Three hundred thirty nine thousand three hundred forty two intron intervals were determined from (i) the splicing junctions of the spliced, uniquely or translocation mapping PTs of the de novo transcriptome, (ii) the splicing junctions of spliced PTs of the genome-guided assembly, and (iii) splice junctions supported by at least 3 uniquely mapped, spliced RNA-seq reads. The two assemblies were filtered to remove potential “contaminants” and “transcriptional noise” in the following order: (i) PTs with top megaBLAST hits on sequences derived from mitochondrial genomes or transcriptomes were excluded because the pig mitochondrial genome has already been well-annotated [50]; (ii) unspliced intronic PTs were discarded; (iii) PTs from the de novo assembly with top megaBLAST hits on sequences from non-vertebrates in the NCBI NT database and without better scored alignments against the two pig reference genomes were filtered out; (iv) unspliced non-intronic PTs within genomic regions for which the maximal CPB was below 50× were removed; (v) multi-mapping and non-mapping PTs from the de novo assembly without significant hits or with non-RNA sequences as top megaBLAST hits in the NT database were excluded.

### Comparing and integrating the de novo and genome-guided transcriptome assemblies

Unspliced non-intronic PTs and spliced PTs in the genome-guided transcriptome that remained after removing “transcriptional noise” were compared to their counterparts in the de novo assembly. For unspliced non-intronic PTs, the comparison was simply done by using the Bedtools *intersect* utility (v2.26.0) [48] to look for overlapping PTs from both assemblies without considering strandedness, which was difficult to determine. On the other hand, because the strandedness of PTs assembled by de novo Trinity could be wrong, the sense strand of the spliced transcripts was first identified based on the splice site consensus sequences, as determined by aligning them to the USMARCv1.0 assembly using GMAP (version 2016-09-23) [49]. Then, the Bedtools *intersect* utility (v2.26.0) [48] and custom Perl scripts were used to find intersections between the spliced PTs from the de novo assembly and from the genome-guided assembly from the same sense strands. Spliced PTs from the de novo assembly that did not overlap any spliced PT from the genome-guided assembly were considered unique to the de novo assembly, and vice versa. Transcripts from the same sense strand that shared at least one intron or exon were considered to arise from the same gene.

The integrated transcriptome included (i) all spliced PTs from the de novo assembly; (ii) spliced PTs that were unique to the genome-guided assembly; (iii) unspliced non-intronic PTs from the filtered de novo assembly; (iv) unspliced non-intronic PTs from the filtered genome-guided assembly that did not overlap counterparts in the de novo assembly; and (v) multi-mapping or non-mapping PTs from the de novo assembly that survived the filtering processes. The BLASTX results were then used to annotate these transcripts with gene ontology (GO) terms, Enzyme Commission (EC) codes, and KEGG (Kyoto Encyclopedia of Genes and Genomes) pathways, by using BLAST2GO with default settings [51, 52].

### Similarity analysis of porcine blood transcripts to their homologous transcripts in humans

Human transcripts were downloaded from the GENCODE database (v25) [53]. Reciprocal DC-megaBLAST was conducted to find the putative homologous transcripts of pigs in humans [54]. Only the best reciprocal BLAST hits with E-value ≤ 10^−20^ were considered significantly similar.

### Validation of the PTs with PacBio IsoSeq full-length cDNA reads

IsoSeq full-length cDNA sequences are assembly-free, so they can be considered as gold standards to validate the PTs from a transcriptome assembly, especially when the genome annotation is poor. Transcriptomes of three tissues (liver, thymus and spleen) of a single cross-bred pig, from which the USMARCv1.0 pig reference genome was assembled, were sequenced by using the IsoSeq technology (H. Liu and T. Smith et al., unpublished). The resulting full-length, non-chimeric reads were error-corrected with preprocessed Illumina RNA-seq reads from the same RNA samples as those that were used for the IsoSeq. A detailed description of IsoSeq and error-correction processes is available in Additional file 1: Supplementary Methods. The 448,771 error-corrected IsoSeq full-length cDNA reads were first aligned to the USMARCv1.0 reference genome using GMAP (version 2016-09-23) [49] and the splice status and the sense strand of the reads were determined as above. To exclude potential genomic DNA contamination from the data, only the spliced, uniquely mapping transcripts from both the integrated transcriptome and IsoSeq reads were compared by using Bedtools (v2.26.0) [48] and custom Perl scripts, after determining the correct sense strands. Transcripts that shared the same exon-intron structures were considered identical. A PT was considered to be fully validated if we found that it had the same exon-intron junctions as at least one IsoSeq full-length cDNA read.

### Assessing completeness of the 5′ termini of the PTs

Published CAGE data from pig macrophages [21] were reanalyzed to estimate the proportion of PTs that have complete 5′ ends. Briefly, adapter sequences and low quality bases were removed from the raw reads using Trimmomatic (v0.32) [42] with following settings: *ILLUMINACLIP:adapters.fa:2:30:10:1:true LEADING:3 TRAILING:3 SLIDINGWINDOW:4:10 LEADING:3 TRAILING:3 MINLEN:20*. Then, the trimmed reads were mapped to the USMARCv1.0 reference genome using STAR (v2.4.1a) [45]. The R/Bioconductor package CAGEr (v1.14.0) [55] was used to obtain transcription starting site (TSS) clusters, as described in its documentation. Sequences of 50 bases that flanked the dominant TSS of each TSS cluster were assumed to be proximal promoters. In addition, the pig genomic regions that matched the mouse and human promoters were retrieved from SSC10.2 based on the published information [21] and realigned to the USMARCv1.0 assembly by using GMAP (version 2016-09-23) [49]. All mapped human and mouse promoters plus the pig promoters identified in macrophages by CAGE were used to evaluate completeness of the 5′ termini of the spliced, uniquely mapping blood PTs from the integrated transcriptome assembly by using the Bedtools *closest* utility (v2.26.0) [48] in consideration of strandedness of PTs and orientations of promoters. A PT was assumed to have a complete 5’ terminus, if a promoter overlapped the first exon or the 500 base pairs (bp) of the 5’ extremity of the first exon when the first exon was longer than 500 bp.

### Comparison of PTs in the integrated transcriptome assembly with the annotated pig transcripts in SSC10.2 and pig RefSeq mRNA sequences

Thirty thousand five hundred eighty five transcripts annotated in the Ensembl SSC10.2 (Ensembl transcripts) and 47,439 RefSeq mRNA sequences curated by the NCBI were separately aligned to the USMARCv1.0 assembly using GMAP (version 2016–09-23) [49]. Only uniquely mapping spliced PTs from the integrated transcriptome assembly were compared to their counterparts from the Ensembl transcripts on the same sense strands by using Bedtools (v2.26.0) [48]. The spliced PTs that did not overlap any spliced Ensembl transcripts were considered as novel transcripts. PTs that shared at least one intron or exon with the Ensembl transcripts from the same sense strand were considered to originate from the same gene. Similarly, a comparison was conducted between uniquely mapping spliced PTs and pig RefSeq mRNAs. Gene loci for PTs were named after the HGNC (HUGO Gene Nomenclature Committee) gene symbols or Ensembl gene identifiers of the transcripts of SSC10.2 and pig RefSeq mRNAs if they were considered to originate from the same gene loci.

### Tissue or cell type origin of PTs in the integrated transcriptome

Lists of human genes specifically or preferentially expressed in given tissue or cell types were downloaded from the CTen (cell type enrichment) database [56]. By assuming porcine and human orthologous genes have preferential expression in same tissues or cell types, the cell or tissue origins of the PTs were inferred.

## Results

### A de novo RNA-seq read-based porcine blood transcriptome

Our goal was to provide a transcriptome resource to facilitate blood-based, high-throughput gene expression studies and to simultaneously improve annotation of the pig genome. To this end, we first assembled a de novo porcine blood transcriptome that incorporates a broad range of RNA-seq data from 146 independent libraries from five studies of blood gene expression in 76 pigs of varied genetic backgrounds and physiological status (Table 1). Raw reads composed of 2.14 billion pairs of paired-end and 1.77 billion single-end reads were trimmed to remove adaptor sequences and low quality bases. Additional file 2: Table S1 shows the effect of trimming on each library. The trimmed reads, consisting of 1.97 billion pairs of paired-end and 1.71 billion single-end reads, were further digitally normalized to reduce the cost and runtime of transcriptome assembly, which resulted in 162.29 million pairs of paired-end and 183.17 million single-end reads. The length distribution of the normalized and trimmed reads is shown in Additional file 3: Figure S1, with most reads of 49, 51 or 100 bases in length. Subsequently, the normalized reads were assembled into putative transcripts (PTs), by using de novo Trinity [44] (Additional file 4: Figure S2). The raw de novo transcriptome assembly included 490,209 PTs that were longer than 200 bases, which potentially originated from 397,560 putative genes (PGs). Additional file 5: Figure S3A shows the length distribution of these PTs; the longest PT was of 22,969 bases. PLEK [47] prediction indicated that the assembly potentially included 56,973 protein-coding PTs and 433,236 PTs without open reading frames (ORFs) that exceeded the minimum length requirements.

### Assessment, annotation, and filtering the de novo blood transcriptome assembly

The representation of the entire transcriptome predicted by RNA-seq reads in the de novo assembly was assessed by mapping all normalized RNA-seq reads to the de novo assembly. The results showed that 66.2% of normalized reads could be mapped back to the de novo assembly, demonstrating that the de novo assembly is relatively comprehensive.

A de novo transcriptome assembly usually results in many fragmented transcripts [43, 44]. Thus, the de novo transcriptome were assessed from different aspects as summarized in Table 2. To assess the extent to which the assembled PTs were full-length, we first aligned the PTs to the protein sequences in the Swiss-Prot database with BLASTX. Based on the best high-scoring segment pairs (HSPs) of the top hits, the de novo assembly included 22,831 PTs that covered more than 80% of the full length of 10,097 protein sequences in the Swiss-Prot database. If the overall percentage of coverage of a protein sequence by its best matching PT was calculated based on all HSPs between them, then we obtained 35,541 PTs that covered 11,645 protein sequences in the database by more than 80% of their length (Additional file 5: Figure S3B). We also aligned all the PTs to the pig RefSeq mRNA using megaBLAST, resulting in 16,010 PTs that covered more than 80% of the full length of 9228 pig RefSeq mRNAs (7760 genes), which is 19.5% of the 47,439 RefSeq mRNA entries (24,122 genes) (Additional file 5: Figure S3C).

**Table 2:** Summary of assessment of the de novo and integrated transcriptome assemblies

Transcriptome assembly	Type of assessment	Purpose	Reference data	Software	Results
The de novo transcriptome assembly	RNA-seq read representation of the assembly	To determine representation of RNA-seq reads	Normalized RNA-seq reads	Trintiy [43, 44]	66.2% of normalized RNA-seq reads could be mapped back to the de novo assembly
	Representation of full-length assembled protein-coding transcripts	To assess the number of full-length PTs	All protein sequences in the Swiss-Prot database	BLASTX [83]	22,831 (nearly) full-length PTs covered more than 80% of the full length of 10,097 protein sequences in the Swiss-Prot database
	Representation of full-length assembled transcripts	To assess the number of full-length PTs	NCBI pig RefSeq mRNAs	DC-megaBLAST [83]	16,010 (nearly) full-length PTs covered more than 80% of the full length of 9228 pig RefSeq mRNAs
	Origin of assembled transcripts	To assess whether the assembled PTs were of porcine genomic origin	Pig reference genomes: SSC10.2 and USMARCv1.0	GMAP [49]	94.2% and 99.4% of the PTs could be mapped to SSC10.2 and USMARCv1.0, respectively
	Similarity-based assessment	To annotate the assembled PTs with known sequences of significant similarity	Sequences in the NCBI NT and NR databases	DC-megaBLAST and BLASTX [83]	69.42% and 21.9% of the PTs shared significant similarities to sequences in the NCBI NT and NR databases, respectively
The integrated transcriptome assembly	Similarity-based assessment	To annotate the assembled PTs with known sequences of significant similarity	Sequences in the NCBI NT and NR databases	DC-megaBLAST and BLASTX [83]	~90% and 63% of the PTs shared significant similarities to sequences in the NCBI NT and NR databases, respectively
	Correctness of exon-intron splicing junctions of PTs	To validate the exon-intron splicing junctions of PTs	Porcine IsoSeq full-length cDNA read data from the liver, spleen and thymus, SSC10.2 transcripts and NCBI RefSeq mRNAs	Bedtools [48] and custom Perl scripts	15,303 PTs and 106,483 IsoSeq sequences had the same exon-intron junctions; and 63,845 uniquely mapping, spliced PTs shared at least one intron or exon with 390,943 IsoSeq reads; 4155 and 6641 PTs shared the same exon-intron junctions as 4010 SSC10.2 annotated transcripts and 6418 RefSeq mRNA sequences, respectively; 54,402 and 60,180 PTs shared at least one intron or one exon with 18,437 SSC10.2 transcripts and 33,870 RefSeq mRNA sequences, respectively
	Completeness of 5′ termini of PTs	To validate the completeness of 5′ termini of PTs	FANTOM5 CAGE data for humans and mouse, and porcine macrophage CAGE data	CAGEr [55], Bedtools [48] and custom Perl scripts	Completeness of the 5′ termini of 37,569 PTs were verified by 43,845 proximal promoters determined by CAGE data
	Length extension of existing transcripts	To determine to what extent the assembled PTs improved over the existing porcine annotation	SSC10.2 transcripts and NCBI pig RefSeq mRNAs	Bedtools [48] and custom Perl scripts	12,262 PTs had both longer 5′ and 3′ termini than the maximally overlapping SSC10.2 transcripts; 9764 PTs had only longer 3′ termini; and14,650 PTs had only longer 5′ termini
	Novelty of PTs	To determine novel PTs	SSC10.2 transcripts and NCBI pig RefSeq mRNAs	Bedtools [48] and custom Perl scripts	41,838 and 35,738 spliced PTs that did not overlap any spliced, uniquely mapping SSC10.2 transcripts or with any spliced, uniquely mapping pig RefSeq mRNA sequence were potential novel transcritps relative to the two reference sets, respectively

Whether the PTs were derived from the pig genome and whether they were “transcriptional noise” was initially determined by mapping them to the two available pig reference genomes, SSC10.2 and USMARCv1.0 (Additional file 4: Figure S2). This analysis indicated that 94.2% and 99.4% of the PTs could be mapped to SSC10.2 and USMARCv1.0, respectively, suggesting that nearly all of the PTs were of porcine origin and the USMARCv1.0 assembly had better representation of the pig genome. This result led us to rely on the USMARCv1.0 reference genome whenever a pig reference genome was used. As summarized in Additional file 4: Figure S2, mapping the PTs on the USMARCv1.0 reference genome identified 115,862 spliced and 359,012 unspliced PTs among the uniquely or translocation mapping PTs. Among the unspliced PTs, 331,774 (92.4%) PTs overlapped intron intervals, most of which were likely intronic sequences that were detected by RNA-seq. We also identified 19,184 unspliced non-intronic PTs which were mapped to genomic regions for which the maximal coverage per base (CPB) by the normalized RNA-seq reads was below 50×. We designated these unspliced intronic PTs and unspliced non-intronic PTs mapped to genomic regions of relative low CPB collectively as “transcriptional noise”, which were to be removed from the de novo assembly.

An additional screen to identify non-porcine contaminants among the PTs was performed by searching for sequences with significant similarity to the PTs in the NCBI NT database using DC-megaBLAST. We found 340,286 (69.42%) PTs with significant top DC-megaBLAST hits on sequences from the NT database (Additional file 4: Figure S2). There were also 2567 (0.5%) PTs that had top BLAST hits on sequences from non-vertebrate species, including bacteria, plants, fungi, and viruses, and no better alignment against the pig reference genomes. These 2567 PTs very likely did not belong to the blood transcriptome. Thus they were to be removed from the de novo assembly. The species distribution of the top BLAST hits on sequences in the NT database is shown in Additional file 5: Figure S3D. The distributions of percentage of identity, percentage of query coverage, bit score and E-value of top BLAST hits are shown in Additional file 5: Figure S3E. The biotypes of the top hits were mRNA/cDNA, ncRNA, misc_RNA, genomic DNA and mitochondrial DNA for 114,937, 5736, 10,970, 206,024 and 52 PTs, respectively (Additional file 4: Figure S2).

The PTs were also aligned to protein sequences curated in the NCBI NR database using BLASTX to get protein-level annotation. This analysis identified 107,237 (21.9%) PTs whose putative proteins had significant similarity to sequences in the NR database, with E-values ≤10^−6^. The species distribution of the top BLASTX hits in the NR database is shown in Additional file 5: Figure S3F; over half were similar to pig protein sequences. The distributions of percentage of identity, percentage of query coverage, bit score and E-value of top BLASTX hits are shown in Additional file 5: Figure S3G, and show that, on average, 38% of the length of PTs were covered by the cognate proteins in the database and the percentage of identity was over 95%.

The de novo transcriptome assembly was filtered to remove mitochondrial genome-derived sequences, “contaminants” and “transcriptional noise” as described in Materials and Methods. The filtered de novo assembly consisted of 126,225 PTs (57,272 PGs) that included 115,859 spliced, uniquely or translocation mapping PTs; 8042 unspliced non-intronic PTs; and 2324 multi-mapping or non-mapping PTs with RNA sequences as top BLASTN hits (Additional file 4: Figure S2).

### Supplementing the de novo assembly with a genome-guided transcriptome assembly

To augment the de novo Trinity assembly, a genome-guided assembly of the blood transcriptome was also performed using Cufflinks with the USMARCv1.0 reference genome as the guide (see schema in Additional file 6: Figure S4). The input was the same normalized RNA-seq reads as was used for de novo Trinity assembly, and the output assembly contained 208,210 PTs, of which 162,294 (78%) were longer than 200 bases (see Additional file 7: Figure S5A for length distribution). The latter included 30,885 spliced and 131,409 unspliced PTs, which included 98,983 intronic PTs and 32,426 non-intronic PTs (Additional file 6: Figure S4). The genome-guided assembly after these steps includes only PTs that were not intronic and longer than 200 bases.

We validated the resulting genome-guided assembly by searching the NT and NR databases for sequences of significant similarity. Of the remaining 63,311 PTs, 53,436 PTs (84.4%) had significantly similar sequences in the NT database (Additional file 7: Figure S5B). The biotypes of the top hits were mRNA, ncRNA, misc_RNA, genomic DNA and mitochondrial DNA for 24,864, 648, 1897, 256,013, and 14 PTs, respectively (Additional file 6: Figure S4). On the other hand, 30,018 PTs (47.4%) potentially encoded protein sequences that were significantly similar to protein sequences in the NR database (Additional file 7: Figure S5C).

Finally, we removed 30,604 unspliced non-intronic PTs that mapped to genomic regions with relatively low CPB by normalized RNA-seq reads, and 14 mitochondrial genome-derived PTs from the genome-guided transcriptome assembly. The filtered genome-guided assembly that was used for comparison to the de novo transcriptome was thus composed of 32,702 PTs, including 30,885 spliced and 1817 unspliced non-intronic PTs (Additional file 6: Figure S4).

### Comparing and integrating the de novo and genome-guided porcine blood transcriptomes

The use of different assembly approaches for the de novo and genome-guided assemblies resulted in significant differences between the predicted transcripts. Integration of the two sets of transcripts was performed as shown in Additional file 8: Figure S6. Of the 115,859 spliced PTs in the filtered de novo assembly, 113,286 PTs had identifiable sense strands. Among the 30,885 spliced PTs in the filtered genome-guided assembly, 24,913 shared at least one intron or exon with 60,702 spliced PTs with identifiable sense strands from the filtered de novo assembly. Of the spliced PTs in the genome-guided assembly, 45 and 8967 PTs had exactly the same exon-intron structures and same exon-intron junctions as 47 and 10,008 spliced PTs, respectively, in the filtered de novo assembly. On the other hand, among the 1817 unspliced non-intronic PTs in the filtered genome-guided assembly, 1086 PTs overlapped 1885 of the 8042 unspliced non-intronic PTs in the filtered de novo assembly.

The final integrated transcriptome was composed of 132,928 PTs, including (i) 115,859 spliced PTs from the filtered de novo assembly; (ii) 5972 spliced PTs unique to the filtered genome-guided assembly; (iii) 8042 unspliced non-intronic PTs from the filtered de novo assembly; (iv) 731 unspliced non-intronic PTs from the filtered genome-guided assembly, which did not overlap counterparts from the filtered de novo assembly; and (v) 2324 multi-mapping or non-mapping PTs with known RNA sequences as BLAST top hits. Based on PLEK [47] prediction, 47,463 PTs (35.7% of total PTs; 40.0% of spliced PTs) potentially encode proteins.

**Fig. 1:**
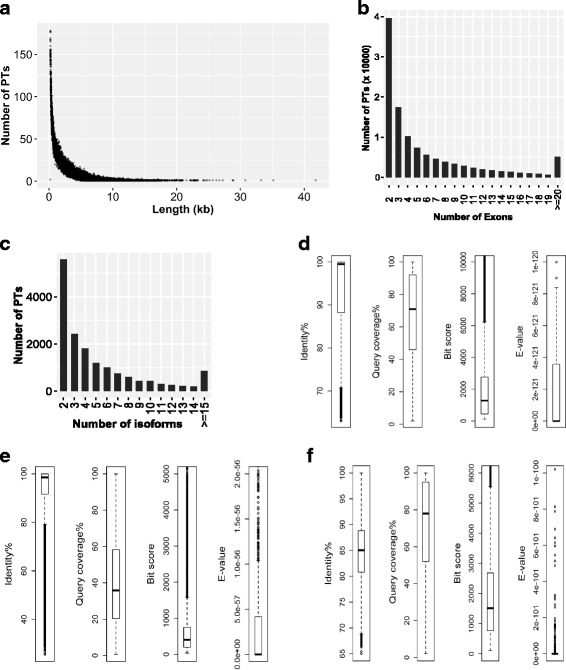
Characterization of the integrated transcriptome assembly. **a** Length distribution of the PTs of the integrated transcriptome; **b** Exon number distribution for the uniquely mapping spliced PTs of the integrated transcriptome; **c** Distribution of the number of isoforms per PT for PTs with at least two isoforms in the integrated transcriptome; **d**, **e** Boxplots showing the distributions of percentage of identity, percentage of query coverage, bit scores and E-values of the top BLAST hits of the PTs in the NCBI NT (**d**) and NR (**e**) databases by using DC-megaBLAST (**d**) and BLASTX (**e**), respectively. **f** Boxplots showing the distributions of percentage of identity, percentage of query coverage, bit scores and E-values of the best reciprocal DC-megaBLAST hits of the PTs in the human transcriptome (GENCODE v25). For clearer visualization, larger outliers of bit scores and E-values are not displayed in D-F

The distributions of the length, number of exons, and number of isoforms of PTs in the integrated transcriptome are shown in Fig. 1a-c. In total, 119,463 (~90%) and 83,828 (63%) of the PTs shared significant similarities to sequences in the NCBI NT and NR databases, respectively. The biotypes of the top megaBLAST hits in the NT database were mRNA/cDNA, misc_RNA, ncRNA and genomic DNA for 80,825, 6904, 1616 and 30,118 PTs, respectively. The distributions of percentage of identity, percentage of query coverage, bit score and E-value of top megaBLAST and BLASTX hits against the NT and NR databases are shown in Fig. 1d and e, respectively.

We then compared the PTs in the integrated transcriptome assembly to the human transcriptome to determine their similarity at the nucleotide level. The distributions of percentage of identity, percentage of query coverage, bit score and E-value of the best reciprocal DC-megaBLAST hits of 19,398 porcine blood PTs on 19,398 human transcripts are shown in Fig. 1f. The percentage of identity between porcine blood PTs and their best human reciprocal DC-megaBLAST hits was above 65%, with a median of 85%.

**Fig. 2:**
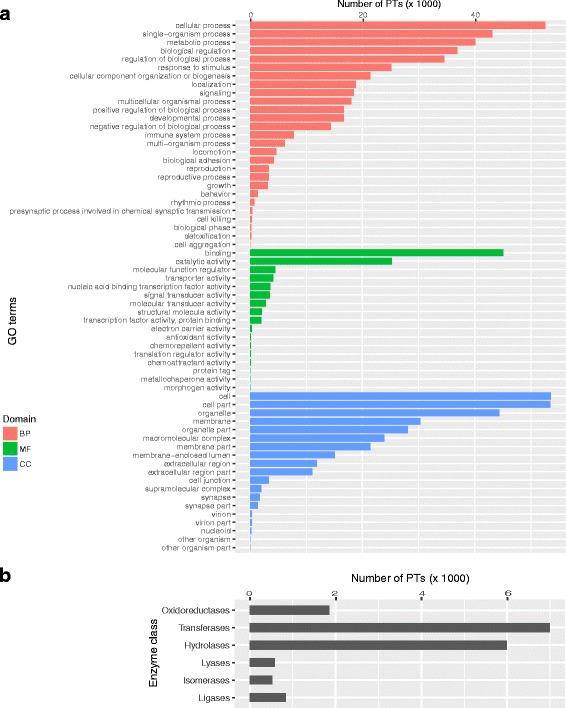
GO terms and EC code annotation of the integrated transcriptome assembly. **a** Distribution of the top 20 GO terms at level two, where available; **b** Distribution of the six main EC classes

The PTs in the integrated transcriptome were annotated with GO terms, EC codes, and KEGG pathways by using BLAST2GO. Schematic summaries of mapping and annotation of GO terms by BLAST2GO are shown in Additional file 9: Figure S7. The distribution of PTs that were annotated with GO terms and EC codes are shown in Fig. 2a and b, respectively. In summary, 68,754 PTs were annotated with 15,965 unique GO terms (Additional file 10: Table S2) and 7618 PTs associated with EC codes were further annotated with 134 KEGG pathways (Additional file 11: Table S3). Consistent with the fact that blood was the source of the RNA samples, a GO-BP term, GO:0002376 (immune system process) ranked as a level-two BP term associated with 7785 PTs (Fig. 2a). More specific immune system process-related GO terms associated with PTs are displayed in Additional file 12: Figure S8. Among them, immune response, innate immune response, and inflammatory response were the top three GO-BP terms that were associated with many PTs. The purine metabolism KEGG pathway was associated with the largest number of PTs, as shown in Additional file 13: Figure S9.

In addition, we inferred the tissues or cell types where the PTs were expressed by assuming humans and pigs share the same lists of genes highly or specifically expressed in given tissues and cell types [56]. Transcripts of porcine genes whose human counterparts highly or specifically expressed in whole blood, T cells, B cells, monocytes, myeloid cells, NK cells, early erythroid, B lymphoblasts, and many other solid tissues were included in the integrated transcriptome assembly (Additional file 14: Table S4).

### Validation of PTs in the integrated blood transcriptome

**Fig. 3:**
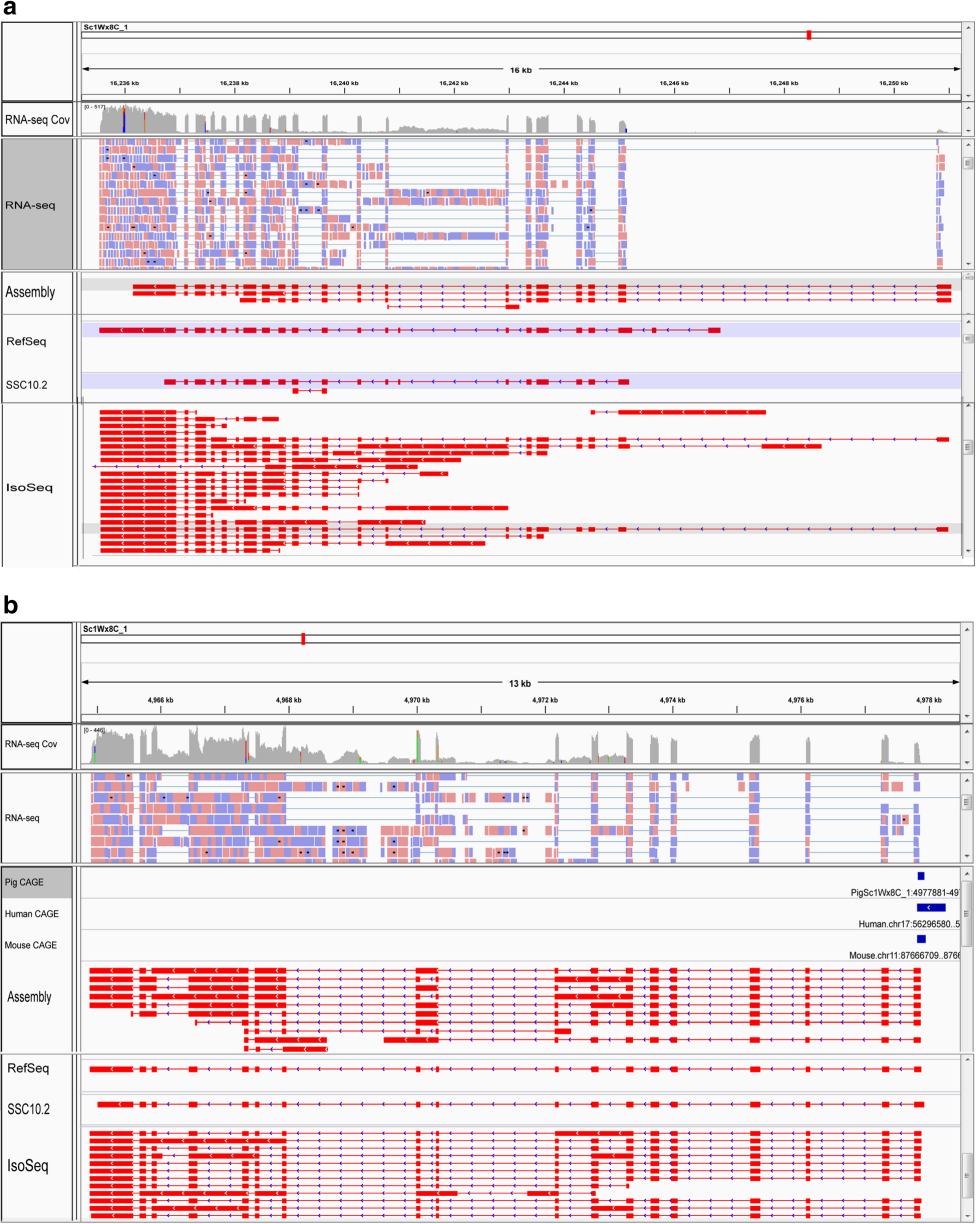
IsoSeq full-length cDNA reads (**a**) and CAGE data (**b**) validate fine structures of PTs in the integrated transcriptome assembly. **a** An example showing one assembled PT shadowed gray in the “Assembly” panel was validated by one IsoSeq full-length cDNA read shadowed gray in the “IsoSeq” panel in terms of intron arrangement. For references, from top to bottom displayed are genomic coordinates, genome coverage by the normalized RNA-seq reads, aligned RNA-seq reads, pig RefSeq mRNAs, SSC10.2 transcripts and IsoSeq read alignments. In the panel labeled as “RNA-seq Cov”, heights of the gray or colored bars represent CPB by the RNA-seq reads. In the “RNA-seq” panel, purple and blue boxes represent reads mapped to the forward and reverse strands of the chromosome; while the thin segments represent introns spanned by spliced reads. In the panels labeled as “Assembly”, “RefSeq”, “SSC10.2” or “IsoSeq”, red boxes represent exons, and thin segments stand for introns, with arrows indicating the orientation of the sense strand. The “Assembly” panel shows PTs mapped to this genomic window. **b** An example showing a conserved proximal promoter among pigs, humans and mice, determined by CAGE, overlaps the 5′ termini of several assembled isoforms of a gene, indicating completeness of the 5′ termini of those PTs. Meaning of the symbols is the same as those in (**a**); in addition, the blue boxes stand for proximal promoters determined by CAGE in porcine macrophage, human and mouse cells in the three panels labeled with “Pig CAGE”, “Human CAGE” or “Mouse CAGE”

Many assembled PTs were validated by mapping the PTs to the reference genomes, and similarity searches in the NT, NR and Swiss-Prot databases, and in the NCBI pig RefSeq mRNA collection. However, due to the complexity of the pig genome and the short length of the RNA-seq reads, RNA-seq read-based transcriptome assembly is error-prone. Common errors include chimerism and artificial splice isoforms. Thus, the integrated transcriptome was validated from different perspectives (see summary in Table 2). To further validate the authenticity of the PTs, we compared 111,002 spliced PTs that uniquely mapped to the pig reference genome and that had identifiable sense strands in the integrated transcriptome to 448,771 error-corrected, assembly-free PacBio IsoSeq full-length cDNA reads with determined sense strands from three porcine tissues: the liver, thymus and spleen (H. Liu, T. Smith et al., unpublished.). We found that (i) 15,303 PTs and 106,483 IsoSeq sequences had the same exon-intron junctions (Addtional file 1: Figure S8A); and (ii) 63,845 uniquely mapping, spliced PTs shared at least one intron or exon with 390,943 IsoSeq reads (See an example in Fig. 3a).

**Fig. 4:**
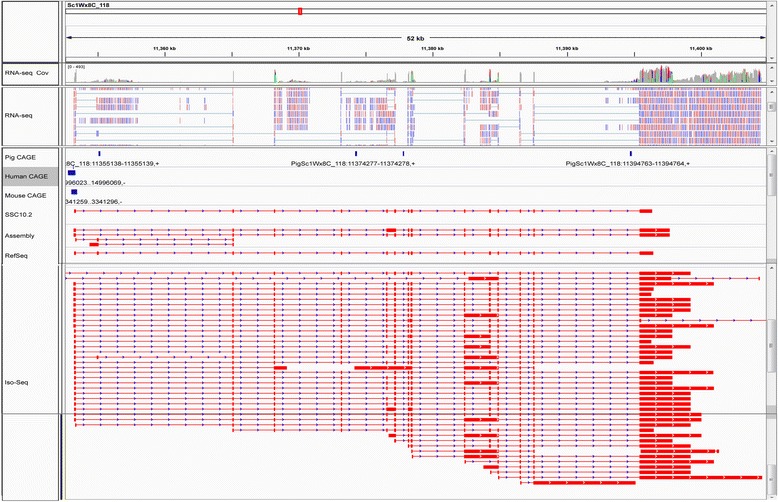
The integrated blood transcriptome assembly improves the structural annotation of the porcine genome compared to the SSC10.2 annotation. The example shown is for the Artemis gene locus, annotation of which in SSC10.2 was improved by extending the 3′ UTR and adding novel isoforms. Meaning of the symbols is the same as those in Fig. 3. The two assembled Artemis isoforms are verified by IsoSeq reads, which show many more isoforms for the Artemis gene. For references, also shown are proximal promoters determined by CAGE data and a RefSeq mRNA of the Artemis gene

IsoSeq reads are generally considered as high-quality transcript standards for evaluating correctness and completeness of gene structural annotation. However, not all IsoSeq transcripts that have switching oligonucleotide and polyA tracts actually have complete 5’ and 3’ UTRs due to various artifacts of the library preparation process. In particular, the switching oligonucleotide that is used to identify the 5’ end may not accurately represent the 5’ terminal cap structure. This type of artifacts is more pronounced for long transcripts, for which the cDNA synthesis may not have reached the 5’ termini. Thus, we used available pig macrophage CAGE data [21] and human and mouse CAGE data from the FANTOM5 project [57] to verify the completeness of 5’ termini of the above-mentioned 111,002 spliced, uniquely mapping PTs. In summary, we found 8529 proximal promoters in the pig macrophage data that were less than 50 bases from the 5’ termini of 19,172 PTs (See examples in Figs. 3b and 4). Based on all promoters identified by CAGE in humans, mice and pigs, we found 43,845 proximal promoters that were less than 50 bases away from the 5’ termini of 37,569 PTs in total. The distribution of distance from the pig macrophage or human/mouse/pig proximal promoters to the 5’ termini of the nearest PTs is shown in Additional file 15: Figure S10A and S10B, respectively.

### Comparison of the putative blood transcripts with SSC10.2 transcripts and pig RefSeq mRNA sequences

The extent to which the integrated transcriptome PTs improved the existing annotation was evaluated by comparing the PTs with the transcript sets of the SSC10.2 and pig RefSeq mRNA collections, after mapping them to the USMARCv1.0 reference genome. To avoid ambiguity, we only compared the 111,002 spliced, uniquely mapping PTs with identifiable sense strands separately to the 23,565 spliced, uniquely mapping SSC10.2 pig transcripts and the 42,931 spliced, uniquely mapping RefSeq mRNA sequences. We found that (i) 4155 and 6641 PTs shared the same exon-intron junctions as 4010 SSC10.2 annotated transcripts and 6418 RefSeq mRNA sequences, respectively; and (ii) 54,402 and 60,180 PTs shared at least one intron or one exon with 18,437 SSC10.2 transcripts and 33,870 RefSeq mRNA sequences, respectively.

**Fig. 5:**
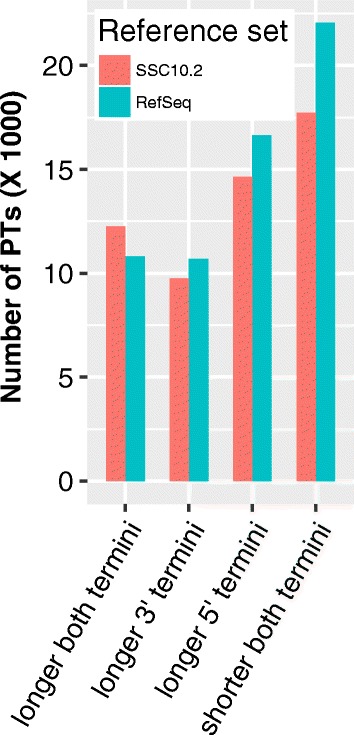
Length comparison between PTs and transcripts in the reference sets. The lengths of uniquely mapping spliced PTs were compared with those of SSC10.2 transcripts and pig RefSeq mRNAs. The number of PTs with longer 5′ and 3′, only longer 3′, and only longer 5′ termini, or neither terminus than their maximally overlapping reference transcripts in SSC10.2 annotation (*red*) and RefSeq mRNA collection (*blue*), respectively is as displayed

Notably, we also found that (1) 12,262 PTs that had both longer 5’ and 3’ termini than the maximally overlapping SSC10.2 transcripts; (2) 9764 PTs had only longer 3’ termini; (3) 14,650 PTs had only longer 5’ termini; and (4) 17,726 PTs had both shorter 5’ and 3’ termini. Similar results were seen when the lengths of PTs was compared with those of RefSeq mRNA sequences (Fig. 5). These length comparisons are shown in Additional file 16: Figure S11A and S11B. A specific example in Fig. 4 shows that PTs that arose from the Artemis gene (DCLRE1C, ENSSSCG00000011049), loss-of-function mutations of which cause SCID disorder in pigs [58], had an extended 3’ UTR and one more isoform relative to the SSC10.2 transcript, ENSSSCT00000012093. In conclusion, a large number of PTs were longer than their maximally overlapping transcripts in the two reference sets, although many PTs were shorter than their maximally overlapping transcripts in the two reference sets. These shorter PTs included both transcript fragments and real intact transcript isoforms which were shorter than their maximally overlapping transcripts in the two reference sets. Gene symbols or Ensembl gene IDs of Ensembl SSC10.2 transcripts or pig RefSeq mRNA sequences were assigned to PTs arising from the same gene loci (Additional file 14: Table S4). Interestingly, there frequently was a one-to-one relationship between overlapping PTs and pig RefSeq mRNAs; but in many cases, one PT corresponded to more than one overlapping SSC10.2 transcript, which might indicate some of these SSC10.2 transcripts were actually fragments of long transcripts.

In addition, we also found 41,838 and 35,738 spliced PTs that did not overlap any spliced, uniquely mapping SSC10.2 transcripts or with any spliced, uniquely mapping pig RefSeq mRNA sequences, respectively, by these comparisons. These PTs were potentially novel transcripts relative to the corresponding reference sets of transcripts used for comparison. The distribution of exon number of the two sets of potentially novel PTs is as shown in Additional file 16: Figure S11C.

## Discussion

We assembled a comprehensive, verified transcriptome of porcine peripheral blood by using large amounts of RNA-seq short reads from multiple independent studies with pigs of different genetic backgrounds and physiopathological conditions. The final transcriptome, consisting of 132,928 PTs, was mainly composed of 126,225 PTs from the de novo transcriptome assembly, supplemented with 6703 PTs from the genome-guided assembly. We discuss below our choices that led to this large number of PTs, which centered on maximizing the discovery of genes expressed in porcine whole blood, while minimizing the inclusion of contamination and technical artifacts. Primarily, we focus on two aspects of these results: (1) possible reasons why we obtained such a large number of assembled transcripts, and (2) our approaches to controlling the quality and validating the accuracy of these transcripts.

### Why so many assembled PTs?

The most recent version of GENCODE (version 25) annotated 58,037 genes for the human genome, including 19,950 protein-coding genes, 15,767 long noncoding RNA genes, 7258 small noncoding RNA genes, and 14,650 pseudogenes [59]. These genes are associated with 198,093 transcripts, which include a wide range of transcript types [53, 59]. Given the similarities of the porcine genome to that in human, we assume porcine and human genomes encode similar numbers of genes and transcripts. Thus we predict that this number of assembled PTs is much higher than the actual, yet unknown, number of expressed transcript isoforms in pig blood. We consider five sources that may have contributed to the high number of assembled PTs as follows.

- Intermediate splicing by-products and genomic DNA contamination

More than 500 million normalized reads, representing more than 6 billion raw reads, were used to create both the de novo and genome-guided transcriptome assemblies. We took this assembly strategy because it has been shown that this strategy can significantly reduce the runtime and cost of transcriptome assembly and increase the chance of gene discovery when the post-normalization *k*-mer coverage was no less than 50× [43]. Cellular transcripts have a very broad range of abundance, such that some transcripts are so abundant that the other lowly expressed transcripts have a vanishingly small chance to be sequenced in a routine RNA-seq run, which makes it difficult to confidently reconstruct transcripts of such low expression levels from the resulting RNA-seq reads. In addition, gene expression depends on genetic background, developmental stage, and internal and external conditions. By combining large amounts of RNA-seq reads from diverse studies that sampled animals with different genetic backgrounds, ages and treatment conditions, we alleviated the issue of poor read coverage of lowly expressed genes and increased the chances of detecting expressed genes.

However, a side effect of this strategy was that it increased the occurrence of “transcriptional noise” and genomic DNA contamination in the resulting assembly. For example, more than 67.7% and 61.0% of the PTs from the de novo and genome-guided assemblies, respectively, were unspliced and overlapped intron intervals. These might mainly arise from incompletely processed RNA or from spliced but not yet decayed intronic fragments, although most nascent transcription is co-transcriptionally spliced [60]. The portion of unspliced PTs resulting from DNA contamination as discussed in [61–63] is probably small, because we saw many more intragenic unspliced PTs than intergenic unspliced PTs, although the genic portion of the genome is much smaller than the intergenic portion. Although RNA samples usually undergo DNase I digestion and oligo-dT-coated bead selection, it is possible that some genomic DNA fragments carried over into library construction [61–63]. One interesting finding was that there was a considerable number of unspliced PTs that had at least one stretch (> 20) of A’s or T’s in their sequence, which suggested some genomic fragments and intronic pieces with a stretch of A’s might have been enriched by oligo-dT bead capture. In addition, given the dynamics of RNA biogenesis, for highly expressed genes, it is possible that some nascent transcripts containing intronic sequences due to incomplete splicing were captured during library construction and eventually sequenced, although they were poly (A)-free. Also, by visualizing the reads aligned to the reference genome in IGV [64], we found that often a long stretch downstream of the 3’ termini of highly expressed transcripts was transcribed and sequenced. This might be due to alternative polyadenylation sites or because termination of transcription of RNA polymerase II occurred downstream, far away from the polyadenylation site. Reads from these regions often assembled into several fragmented PTs, which could account for a significant portion of the unspliced non-intronic PTs. Sequencing of the porcine blood transcriptome by using PacBio IsoSeq will help determine the real scenarios.

b) Some assembled sequences are from sources other than the porcine genome

Another side effect of using normalized reads from large amounts of raw RNA-seq data was that it increased the chance to detect contamination of non-pig genome origins. For example, we assembled 2567 PTs for which a similarity search by using DC-megaBLAST suggested they were likely from feed, parasites, fungi, bacteria and viruses, and had poorer, if any, alignments to the pig reference genomes. These contaminants might have been from sample collection and/or manipulation processes, but could also derive from the microbiome in the peripheral blood of pigs and absorbed dietary RNA from feed the pig consumed. Traditional microscopy and current deep sequencing technology have clearly shown that there are parasites, fungi, bacteria and viruses in the bloodstream in diseased or non-diseased situations in humans [65–68]. As pigs are usually kept in dirty environments, they likely carry some parasites, fungi and other microbes in their bloodstream, which could come from blood exposure to the oral cavity, respiratory or intestinal tracts, although they may not show clinical symptoms. Notably, chronic inflammatory diseases can increase the chance for gut microbiome to translocate into the circulating blood, where they may be in a dormant state [67]. In two of the five independent studies that were used here, pigs suffered from chronic inflammation due to *Salmonella* infection [35] or chronic LPS stimulation (H. Liu and K. Feye et al. unpublished). In addition, diet-derived miRNAs have been detected in the bloodstream and adipose tissue of animals [69–71]. So it is possible that some amount of dietary RNA could survive in the circulating blood in pigs, especially when gut integrity is compromised during inflammation.

c) PTs derived from porcine endogenous retroviruses, non-blood exosomal cargos, and beyond

Another source of PTs of non-blood origins is endogenous retroviruses in pigs. It is known that a variety of endogenous retroviruses can be active in pigs [72–74]. We identified 61 PTs derived from pig endogenous retroviruses in the de novo assembly. Furthermore, exosomal RNAs from non-blood cells and non-exosomal, cell-free RNAs in circulating blood, which have been identified in humans [75, 76], could further diversify the apparent peripheral porcine blood transcriptome.

d) Fragmented transcripts due to technical limitations

Another reason why so many PTs were assembled was that de novo transcriptome assembly based on RNA-seq reads tends to result in fragmented transcripts due to sequencing errors, genetic polymorphisms, uneven read coverage, and repetitive sequences. In this study, sequencing adapters and low quality bases were removed from reads and trimmed reads were digitally normalized before assembly, which can partly alleviate problems caused by sequencing errors and uneven read coverage for most transcribed genes in circulating blood. However, the use of RNA-seq reads from multiple studies with pigs of different genetic backgrounds could aggravate the issue of genetic polymorphisms, which are more common in non-coding regions, such as UTRs. In fact, we found that many 3’ UTRs, which were well-covered by reads, were assembled into fragments separate from the corresponding coding regions (data not shown). In addition, a small fraction of repetitive sequences in the mature transcripts, as evidenced by multiple mapping RNA-seq reads, can contribute to fragmented assembly of some transcripts.

e) Complexity of the transcription landscape in the pig genome

Lastly, the large number of PTs in the transcriptome assembly can also be due to the complexity of the landscape of eukaryotic transcriptomes [59, 77, 78]. One hundred ninety eight thousand ninety three transcripts associated with 58,037 genes have been annotated for the human genome (GENCODE v25) [53]. Given the similarities between the pig and human genomes, it is very possible that the transcription landscape of the pig genome is also very complex and significantly underestimated in the current pig genome annotation [20]. Our de novo assembled blood transcriptome at least partly supports this inference, although it might overestimate the total number of transcripts due to technical limitations of de novo assembly as discussed above.

The initial transcriptome assemblies were filtered by mapping the PTs to reference genomes or by comparison to existing transcript databases, to remove PTs which were (i) most likely not derived from the pig genome, (ii) genomic DNA contamination or (iii) intermediate splicing transcription products. These filtering steps may remove some true porcine transcripts that happened to be mono-exonic and derived from intronic regions, such as intronic ncRNAs, or that by chance had high similarity to non-vertebrate nucleic acid sequences. However, on balance, such filtering is appropriate and conservative, and we believe that, as the majority of protein-coding and long ncRNA transcripts are spliced [79], the few true porcine transcripts that were removed by this procedure is outweighed by the potential for a large number of false transcripts that may be included in the assembly without filtering.

### How accurate and comprehensive was the assembled final transcriptome?

Short RNA-seq read-based transcriptome assembly usually results in a large portion of transcript fragments, as discussed above, thus leading to overestimation of the number of transcripts or genes in the target tissue or cell. To assess the quality of the integrated transcriptome assembly, which mainly consisted of PTs from the de novo assembly, we evaluated the assemblies in various ways, which led to three major conclusions.

- The de novo assembly represented a majority of the normalized blood RNA-seq data, and covered nearly the full length of many curated protein sequences, and porcine RefSeq mRNAs.

More than 66% of total input RNA-seq reads could be mapped to the de novo transcriptome, which was comparable to those reported mapping rates (65–85%) for other similar assemblies [80]. In addition, we verified 35,541 and 16,010 PTs that covered more than 80% of the full length of the Swiss-Prot protein and pig RefSeq mRNA sequences, respectively. Note that this may underestimate the number of full-length PTs, as the transcriptome and proteome for porcine tissues are poorly annotated.

b) The short read-based transcriptome assembly was substantially verified by PacBio IsoSeq long-read data, CAGE data, as well as by existing porcine gene annotations.

A global sequence alignment-based method for assessing the quality of the assembled transcriptome may not reveal some structural details of the assembled transcripts, such as correctness of the exon-exon junctions of the isoforms and completeness of transcript ends. Therefore, we used the assembly-free, PacBio IsoSeq long cDNA reads from the three porcine tissues (liver, thymus and spleen), which were the IsoSeq data available, to validate the correctness of the fine structures of the PTs. These non-blood sources of cDNA reads are applicable because (i) different tissues usually share a significant portion of expressed transcripts, (ii) all three tissue biopsies contained some blood, (iii) the thymus and spleen are primary immune tissues, with substantial proportions of cells found in blood such as monocytes and lymphocytes [81]. We found that 15,311 PTs (14% of the PTs in the integrated transcriptome assembly) and 106,484 IsoSeq reads shared the same intron arrangement. However, some peripheral blood-specific transcripts may not be validated by these PacBio IsoSeq reads. In addition, we made use of the extant human and mouse CAGE data from the FANTOM5 project [57] as well as pig macrophage CAGE data [21], to check the completeness of the 5′ extremities of the PTs. We found that 37,569 PTs had complete or nearly complete 5′ termini. These analyses indicated that many of the PTs were correctly reconstructed in terms of exonic architecture and completeness of 5’ termini. Completeness of the 3’ termini could be further verified by using the 3’ T-fill sequencing technology [82].

Comparison of PTs in the integrated transcriptome to SSC10.2 annotation identified 4155 PTs with shared intron arrangements to 4010 annotated transcripts, representing 17% of all SSC10.2 transcript annotations. Further, we found that 12,262 PTs had both longer 5’ UTRs and longer 3’ UTRs than the maximally overlapping counterparts in the SSC10.2 annotation. Notably, we also found 41,838 novel spliced PTs that were missing in the SSC10.2 annotation. Similar results were found when the PTs in the integrated transcriptome were compared to the pig RefSeq mRNA set. This suggests that our PTs were useful in expanding the annotation of the pig reference genomes.

c) The integrated transcriptome was well-annotated functionally.

Functional annotation of a transcriptome on a large scale currently depends mainly on sequence similarities between novel and annotated sequences, often across species. By searching the NCBI NT and NR databases, we identified significantly similar sequences for 119,463 (~90%) and 83,828 (63%) PTs, respectively, of the integrated transcriptome assembly; 80,825 (~61%) PTs were potentially mRNA, while the others were potentially ncRNA, misc_RNA, or artifacts. PTs which encoded putative proteins significantly similar to protein sequences in the NR database were further annotated with GO terms, EC codes and KEGG pathways. In total, 68,754 (82%) and 7618 (9%) of the PTs with significant BLASTX hits were annotated with 15,965 unique GO terms and 134 KEGG pathways, respectively. We found that a level-two GO-BP term, immune system process, and many more specific terms related to the immune system process were well represented by PTs in the integrated transcriptome, consistent with the whole blood origin of the RNA-seq data.

## Conclusions

We constructed a comprehensive peripheral blood transcriptome for the pig by combining de novo and genome-guided assemblies of extensive RNA-seq data from multiple experiments. The resulting transcriptome has been carefully filtered to maximize useful information content, validated on a large scale, and well-annotated by using a series of methods. The transcriptome had significant improvement over the SSC10.2 annotation and the pig RefSeq mRNAs. Thus, the transcriptome we assembled can be used to analyze data from future blood-based high throughput gene expression studies and will help more fully annotate the newly assembled pig reference genomes.

## Additional files

Additional file 1: Supplementary Methods. (DOCX 30 kb)

Additional file 2: Table S1. Trimming effect on the raw RNA-seq data. (XLSX 27 kb)

Additional file 3: Figure S1. Length distribution of the normalized trimmed RNA-seq reads used for the porcine blood transcriptome assembly. (PDF 4 kb)

Additional file 4: Figure S2. Flowchart for de novo blood transcriptome assembly, annotation and filtering. The diagram shows the steps involved in construction and filtering of the de novo assembly, and includes the number of PTs that resulted from each step, where appropriate. Refer to the Materials and Methods section for details. Quality of the raw RNA-seq reads for each library was first checked with FASTQC. Subsequently, sequencing adaptors and low quality bases were trimmed from the raw reads. These trimmed reads were then digitally normalized to reduce *k*-mer redundancy. Normalized reads were assembled into putative Trinity transcripts (PTs), which are collectively called “de novo transcriptome assembly”. This assembly was then analyzed in several ways. First, the coding potentials of the PTs were predicted by using PLEK, with PTs of coding potentials higher than zero considered as potentially protein-coding. Then all PTs were separately aligned to the two pig reference genomes, USMARCv1.0 and SSC10.2, by using GMAP. Finally, PTs with significant BLAST hits in the NCBI NT and NR databases were determined by using DC-megaBLAST and BLASTX, with E-value cutoffs of 10^−20^ and 10^−6^, respectively. Because the alignment frequency of the PTs to the USMARCv1.0 reference genomes was much higher than to the SSC10.2 assembly, the de novo transcriptome was filtered based on the USMARCv1.0 mapping results. PTs with top megaBLAST hits on sequences from non-vertebrates and without better alignments with the two reference genomes were considered as “contaminants” and were filtered out. The potential biotypes of the PTs were determined based on the biotypes of their top megaBLAST hits if available. Other removed PTs were (i) PTs with top megaBLAST hits on sequences of mitochondrial genomes; (ii) unspliced intronic PTs; (iii) unspliced nonintronic PTs mapped to genomic regions of maximal coverage per base (CPB) lower than 50× (low-CPB regions); and (iv) multiple mapping or nonmapping PTs on the USMARCv1.0 assembly without top megaBLAST hits on RNA sequences in the NT database. The final filtered de novo transcriptome assembly was composed of 126,225 PTs. (PDF 498 kb)

Additional file 5: Figure S3. Characterization of the de novo transcriptome assembly. (A) Length distribution of PTs in the de novo transcriptome assembly; (B-C) Full-length assessment by using Swiss-Prot protein (B) and pig RefSeq mRNA (C) sequences as standards. In (B), percentage of coverage of the sequences as standards by PTs were calculated based only on the best high-scoring segment pairs (HSP) (“ungrouped percentage of coverage” calculation method) or based on all HSPs (“grouped percentage of coverage” calculation method) between the two aligned sequences; (D, F) Species distribution of top DC-megaBLAST (D) and BLASTX (F) hits of the PTs in the NT and NR databases, respectively; (E, G) Boxplots showing the distributions of percentage of identity, percentage of query coverage, bit scores and E-values of the top BLAST hits of the PTs in the NCBI NT and NR databases by using DC-megaBLAST (E) and BLASTX (G), respectively. For clearer visualization, larger outliers of bit scores and E-values are not displayed. (PDF 1743 kb)

Additional file 6: Figure S4. Flowchart for genome-guided transcriptome assembly, annotation and filtering. The diagram shows the steps involved in construction and filtering of the genome-guided assembly, and includes the number of PTs that resulted from each step, where appropriate. Refer to the Methods section for details. The raw RNA-seq reads that were preprocessed as above (see the legend of Additional file 4: Figure S2) were mapped to the USMARCv1.0 reference genome by using STAR, and then assembled into PTs by using Cufflinks. PTs of 200 bases or shorter in length were removed from the resulting genome-guided transcriptome assembly before further analysis. Splicing status of the 162,294 PTs was determined and unspliced intronic PTs were discarded. Among the remaining PTs, those with significant BLAST hits in the NCBI NT and NR databases were determined by using DC-megaBLAST and BLASTX, with E-value cutoffs of 10^−20^ and 10^−6^, respectively. The potential biotypes of the PTs were determined based on the biotypes of their significant DC-megaBLAST hits. To complete the filtering, we removed from the remaining PTs: (i) PTs with top DC-megaBLAST hits on sequences originating from mitochondrial genomes; and (ii) PTs mapped to genomic regions of maximal CPB lower than 50× (low-CPB regions). The final filtered genome-guided transcriptome consisted of 32,702 PTs. (PDF 360 kb)

Additional file 7: Figure S5. Characterization of the genome-guided transcriptome assembly. (A) Length distribution of PTs in the genome-guided transcriptome assembly; (B, C) Boxplots showing the distributions of percentage of identity, percentage of query coverage, bit scores and E-values of the top BLAST hits of the PTs in the NCBI NT and NR databases by using DC-megaBLAST (B) and BLASTX (C). For clear visualization, larger outliers of bit scores and E-values are not displayed. (PDF 1877 kb)

Additional file 8: Figure S6. Integration of the de novo and genome-guided assemblies. This diagram shows the steps used to integrate the two assemblies that were described in Figs. 1 and 2, and includes the number of PTs that resulted from each step, where appropriate; the overall goal was to identify those genome-guided assemblies that added information to the de novo assembly. The sense strands of 113,286 spliced PTs from the de novo assembly (Set A) were first determined based on consensus splice site sequences after alignment to the USMARCv1.0 reference genome. The spliced PTs in the filtered de novo transcriptome were then compared to their counterparts in the filtered genome-guided transcriptome (Set B) by using the Bedtools *intersect* utility and custom Perl scripts. If a spliced PT from the de novo assembly shared at least one intron or exon, or all introns and exons with a spliced PT from the genome-guided assembly mapped on the sense strand, then they were considered overlapping or exactly the same, respectively. In addition, unspliced PTs from the de novo and genome-guided assemblies were directly compared, without considering their sense strands, by using Bedtools *intersect* utility. PTs from the de novo transcriptome assembly, which did not overlap PTs from the genome-guided transcriptome assembly were claimed to be de novo transcriptome assembly-specific, and vice versa. The final integrated transcriptome consisted of 132,928 PTs, which included all 126,225 PTs from the filtered de novo transcriptome assembly and 6703 PTs specific to the filtered genome-guided transcriptome, including both 5972 spliced and 731 unspliced PTs. (PDF 337 kb)

Additional file 9: Figure S7. Schematic summary of BLAST2GO mapping and annotation. (A) Distribution of source databases based on which GO terms were mapped to PTs in the integrated transcriptome assembly; (B) Distribution of the number of GO terms mapped to PTs of the integrated transcriptome assembly; (C) Distribution of evidence codes of the annotated GO terms mapped to PTs of the integrated transcriptome assembly; (D) Distribution of the number of GO terms mapped to PTs of the integrated transcriptome assembly; (E) Distribution of GO terms at different levels of the hierarchy of GO terms; (F) Distribution of annotation scores of GO terms. (PDF 110 kb)

Additional file 10: Table S2. PTs annotated with GO terms. (XLSX 3074 kb)

Additional file 11: Table S3. PTs annotated with EC codes and KEGG pathways. (XLSX 428 kb)

Additional file 12: Figure S8. Distribution of GO terms related to immune system process. (PDF 36 kb)

Additional file 13: Figure S9. The purine metabolism KEGG pathway highlighted with enzymes encoded by PTs of the integrated transcriptome. Different enzyme codes are highlighted with different colors. (PNG 88 kb)

Additional file 14: Table S4. PTs assigned with HGNC gene symbols and Ensembl pig Gene IDs and tissue or cell types where their human counterparts specifically or preferentially expressed. (TXT 3006 kb)

Additional file 15: Figure S10. The distributions of the distance between proximal promoters defined by pig macrophage CAGE data (A) or all available human/mouse/pig CAGE data (B) to the nearest 5′ termini of spliced, uniquely mapping PTs in the integrated transcriptome assembly. The vast majority of these distances were less than 50 bp, which suggested that the 5′ termini of many PTs were completely assembled. (PDF 28 kb)

Additional file 16: Figure S11. Comparison of PTs of the integrated transcriptome assembly to the IsoSeq cDNA reads, transcripts annotated in Ensembl SSC10.2 and NCBI pig RefSeq mRNA sequences. (A) Venn diagram showing the number of PTs with their intron arrangements validated by the IsoSeq reads, pig RefSeq mRNA sequences and SSC10.2 transcripts; (B) Distribution of exon number of novel spliced transcripts not found in the SSC10.2 annotation or pig RefSeq mRNA set, which provides good evidence of significant extension of current genome annotation; (C) Length comparison between PTs and their maximally overlapping SSC10.2 transcripts; (D) Length comparison between PTs and their maximally overlapping RefSeq mRNA sequences. (PDF 9912 kb)

## Abbreviations

BAM: Binary alignment/map
BLAST: Basic local alignment search tool
BP: Biological process
CAGE: Cap analysis of gene expression
CC: Cellular component
CIGAR: Concise idiosyncratic gapped alignment report
CPB: Coverage per base
CTen: Cell type enrichment
DCLRE1C: DNA cross-link repair 1C
EC: Enzyme commission
ENA: European nucleotide archive
FANTOM: Functional annotation of the mammalian genome
FTP: File transfer protocol
GO: Gene ontology
GTF: Gene transfer format
HSP: High-scoring segment pair
IACUC: Institutional animal care and use committee
IsoSeq: Isoform sequencing
KEGG: Kyoto encyclopedia of genes and genomes
lncRNA: Long non-coding RNA
LPS: Lipopolysaccharide
MF: Molecular function
miRNA: microRNA
misc_RNA: Miscellaneous RNA
ncRNA: Noncoding RNA
NK: Natural killer
NR: The NCBI non-redundant protein database
NT: The NCBI nucleotide database
ORF: Open reading frame
PG: Putative genes
PT: Putative transcript
RBC: Red blood cell
RNA-seq: RNA-sequencing
SAM: Sequence alignment/map
SRA: The NCBI sequence read archive
SSC10.2: Pig reference genome Sscrofa10.2 version
USMARC: Meat animal research center, United States Department of Agriculture
UTR: Untranslated region
WBC: White blood cell
