# Supplementary material for: A high-quality annotated transcriptome of swine peripheral blood
Source: BMC Genomics. 2017 Jun 24;18:479. doi: 10.1186/s12864-017-3863-7 (PMC5483264; doi:10.1186/s12864-017-3863-7)
Supplement: Supplementary file 1 — Supplementary Methods. (DOCX 30 kb) [file 12864_2017_3863_MOESM1_ESM.docx]

**Supplementary Methods**

**Sequencing the transcriptomes of three porcine tissues by using the PacBio IsoSeq and Illumina RNA-Seq technologies**

The transcriptomes of three tissues (liver, spleen and thymus) from a single cross-bred pig (½ Landrace- ¼ Duroc- ¼ Yorkshire), from which the PacBio long read-based reference genome was assembled by using DNA of lung tissue, was sequenced by the US Meat Animal Research Center (MARC, USDA, Clay Center, NE) using the Illumina NextSeq500 and PacBio RSII platforms for RNA-Seq and IsoSeq, respectively. Total RNA from each of the three tissues was extracted using Trizol reagent (ThermoFisher Scientific) and the provided protocol. Briefly, approximately 100 mg of tissue was ground in a mortar and pestle cooled with liquid nitrogen, and the powder was transferred to a tube with 1 ml of Trizol reagent added and mixed by vortexing. After 5 minutes at room temperature, 0.2 mL of chloroform was added and the mixture was shaken for 15 seconds and left to stand another 3 minutes at room temperature. The tube was centrifuged at 12,000 x g for 15 minutes at 4°C. The RNA was precipitated from the aqueous phase with 0.5 mL of isopropanol. The RNA was further purified with extended DNase I digestion to remove potential DNA contamination. The RNA quality was assessed with a Fragment Analyzer (Advanced Analytical Technologies Inc, IA). Only RNA samples of RQN above 7.0 were used for library construction. PacBio IsoSeq libraries were constructed per the PacBio IsoSeq protocol. Briefly, starting with 3 μg of total RNA, cDNA was synthesized by using SMARTer PCR cDNA Synthesis Kit (Clontech, CA) according to the IsoSeq protocol (Pacific Biosciences, CA). Then the cDNA was amplified using KAPA HiFi DNA Polymerase (KAPA Biotechnologies) for 10 or 12 cycles followed by purification and size selection into 4 fractions: 0.8-2 kb, 2-3 kb, 3-5 kb and > 5kb. The fragment size distribution was validated on a Fragment Analyzer (Advanced Analytical Technologies Inc, IA) and quantitated on a DS-11 FX fluorometer (DeNovix, DE). After a second round of large scale PCR amplification and end repair, SMARTbell adapters were separately ligated to the cDNA fragments. Each size fraction was sequenced on 4 or 5 SMART Cells v3 using P6-C4 chemistry and 6 hour movies on a PacBio RS II sequencer (Pacific Bioscience, CA). Short read RNA-Seq libraries were prepared using TruSeq stranded mRNA LT kits and supplied protocol (Illumina, CA), and sequenced on a NextSeq500 platform using v2 sequencing chemistry to generate 2 x 75 paired-end reads.

**Error-correction and redundancy reduction of PacBio IsoSeq full-length cDNA reads**

The Read of Insert (ROI) were determined by using *consensustools.sh* in the SMRT-Analysis pipeline v2.0, with reads which were shorter than 300 bp and whose predicted accuracy was lower than 75% removed. Full-length, non-chimeric cDNA reads were identified by running the classify.py command. Primer sequences as well as the poly(A) tails were trimmed prior to further analysis. Paired-end Illumina RNA-Seq reads from each tissue sample were trimmed to remove the adaptor sequences and low-quality bases using Trimmomatic (v0.32) [1] with explicit option settings: *ILLUMINACLIP:adapters.fa:2:30:10:1:true LEADING:3 TRAILING:3 SLIDINGWINDOW:4:20 LEADING:3 TRAILING:3 MINLEN:25*, and overlapping paired-end reads were merged using the PEAR software (v0.9.6) [2]. Subsequently, the merged and unmerged RNA-Seq reads from the same tissue samples were *in silico* normalized in a mode for single-end reads by using a Trinity (v2.1.1) [3] utility*, insilico_read_normalization.pl*, with the following settings*: --max_cov 50 --max_pct_stdev 100* *--single*. Errors in the full-length, non-chimeric cDNA reads were corrected with the preprocessed RNA-Seq reads from the same tissue samples by using proovread (v2.12) [4]. Untrimmed sequences with at least some regions of high accuracy in the *.trimmed.fq* files were extracted based on sequence IDs in .*untrimmed.fa* files to balance off the contiguity and accuracy of the final reads.

To reduce the redundancy of the PacBio IsoSeq reads of the three tissues, the error-corrected reads from all three tissues were combined after assigning unique identifiers to them, and mapping them to USMARCv1.0 using GMAP (version 2016-09-23) [5]. The sense strands of the spliced reads were determined on the basis of the splice site consensus sequences. Mapped reads were clustered based on chromosome coordinates and strand information using Bedtools [6] and custom Perl scripts. Reads in each cluster were collapsed if they met the following criteria: (1) they shared compatible intron-exon junctions; (2) they had compatible 5’ termini; (3) they differed in the 3’ termini by less than 100 bases in length ([https://github.com/PacificBiosciences/cDNA_primer/ wiki/](https://github.com/PacificBiosciences/cDNA_primer/%20wiki/)tofu-Tutorial-(optional).-Removing-redundant-transcripts).

**References**

1. Bolger AM, Lohse M, Usadel B: **Trimmomatic: a flexible trimmer for Illumina sequence data**. *Bioinformatics* 2014, **30**(15):2114-2120.

2. Zhang J, Kobert K, Flouri T, Stamatakis A: **PEAR: a fast and accurate Illumina Paired-End reAd mergeR**. *Bioinformatics* 2014, **30**(5):614-620.

3. Grabherr MG, Haas BJ, Yassour M, Levin JZ, Thompson DA, Amit I: **Full-length transcriptome assembly from RNA-Seq data without a reference genome**. *Nat Biotechnol* 2011, **29**:644-652.

4. Hackl T, Hedrich R, Schultz J, Forster F: **proovread: large-scale high-accuracy PacBio correction through iterative short read consensus**. *Bioinformatics* 2014, **30**(21):3004-3011.

5. Wu TD, Reeder J, Lawrence M, Becker G, Brauer MJ: **GMAP and GSNAP for genomic sequence alignment: enhancements to speed, accuracy, and functionality**. In: *Statistical Genomics: Methods and Protocols.* Edited by Mathé E, Davis S. New York, NY: Springer New York; 2016: 283-334.

6. Quinlan AR, Hall IM: **BEDTools: a flexible suite of utilities for comparing genomic features**. *Bioinformatics* 2010, **26**(6):841-842.
