# Supplementary figures and images for: A high-quality annotated transcriptome of swine peripheral blood
Source: BMC Genomics. 2017 Jun 24;18:479. doi: 10.1186/s12864-017-3863-7 (PMC5483264; doi:10.1186/s12864-017-3863-7)

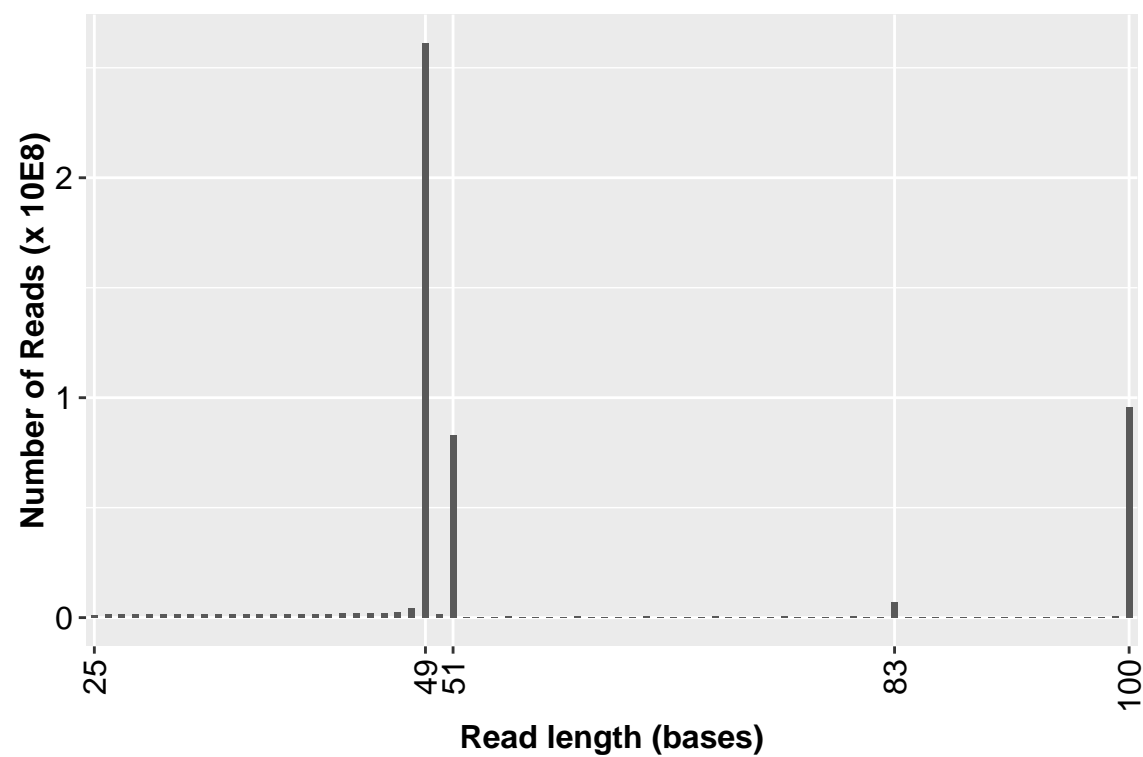

Supplement: Supplementary file 3 — Length distribution of the normalized trimmed RNA-seq reads used for the porcine blood transcriptome assembly. (PDF 4 kb) [file 12864_2017_3863_MOESM3_ESM.pdf]

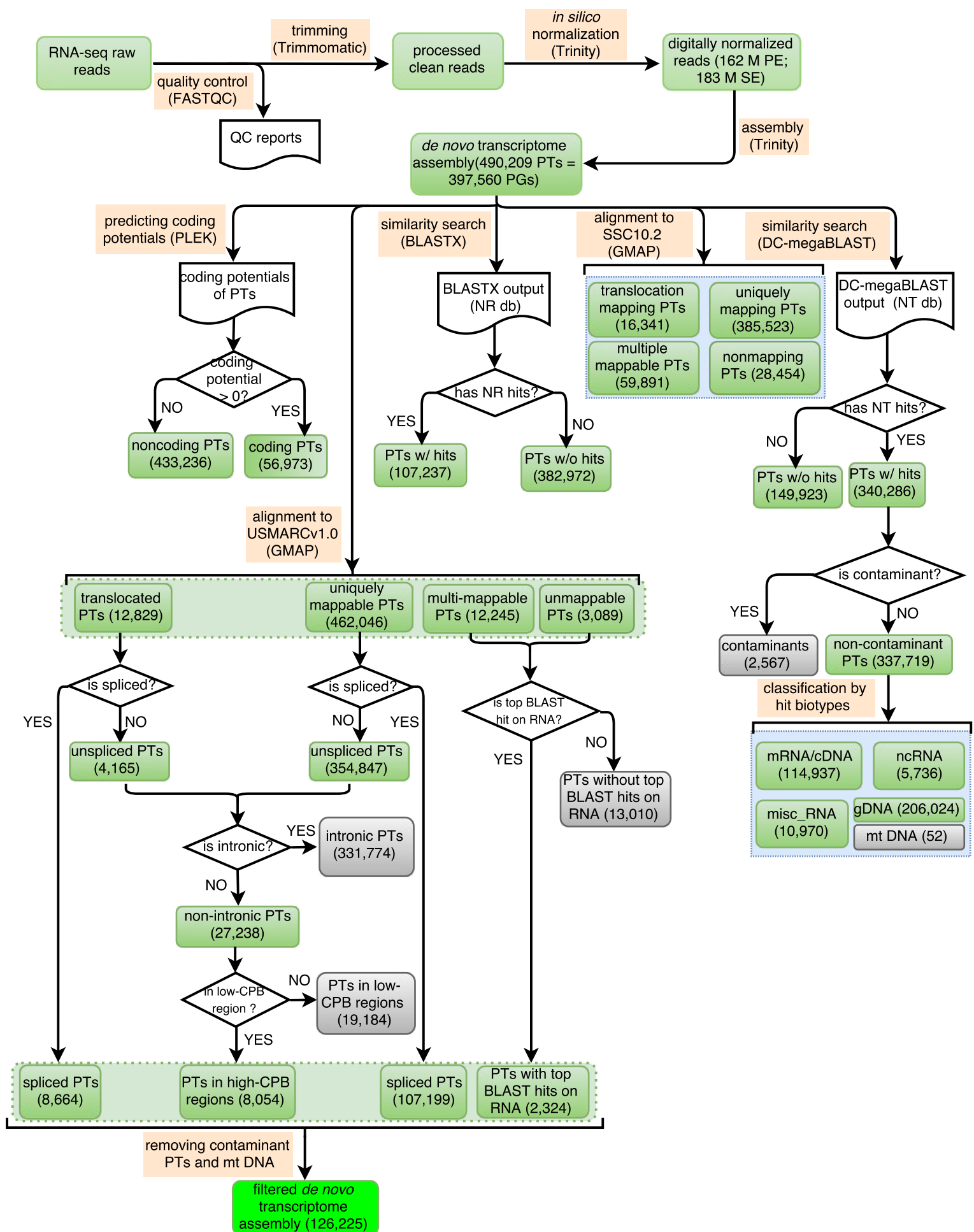

Supplement: Supplementary file 4 — Flowchart for de novo blood transcriptome assembly, annotation and filtering. The diagram shows the steps involved in construction and filtering of the de novo assembly, and includes the number of PTs that resulted from each step, where appropriate. Refer to the Materials and Methods section for details. Quality of the raw RNA-seq reads for each library was first checked with FASTQC. Subsequently, sequencing adaptors and low quality bases were trimmed from the raw reads. These trimmed reads were then digitally normalized to reduce k-mer redundancy. Normalized reads were assembled into putative Trinity transcripts (PTs), which are collectively called “de novo transcriptome assembly”. This assembly was then analyzed in several ways. First, the coding potentials of the PTs were predicted by using PLEK, with PTs of coding potentials higher than zero considered as potentially protein-coding. Then all PTs were separately aligned to the two pig reference genomes, USMARCv1.0 and SSC10.2, by using GMAP. Finally, PTs with significant BLAST hits in the NCBI NT and NR databases were determined by using DC-megaBLAST and BLASTX, with E-value cutoffs of 10−20 and 10−6, respectively. Because the alignment frequency of the PTs to the USMARCv1.0 reference genomes was much higher than to the SSC10.2 assembly, the de novo transcriptome was filtered based on the USMARCv1.0 mapping results. PTs with top megaBLAST hits on sequences from non-vertebrates and without better alignments with the two reference genomes were considered as “contaminants” and were filtered out. The potential biotypes of the PTs were determined based on the biotypes of their top megaBLAST hits if available. Other removed PTs were (i) PTs with top megaBLAST hits on sequences of mitochondrial genomes; (ii) unspliced intronic PTs; (iii) unspliced nonintronic PTs mapped to genomic regions of maximal coverage per base (CPB) lower than 50× (low-CPB regions); and (iv) multiple mapping or nonmapping PTs on the USMARCv1.0 as [file 12864_2017_3863_MOESM4_ESM.pdf]

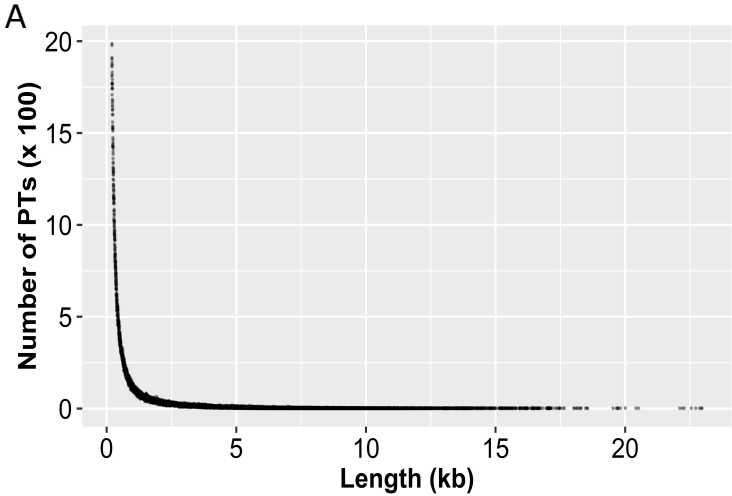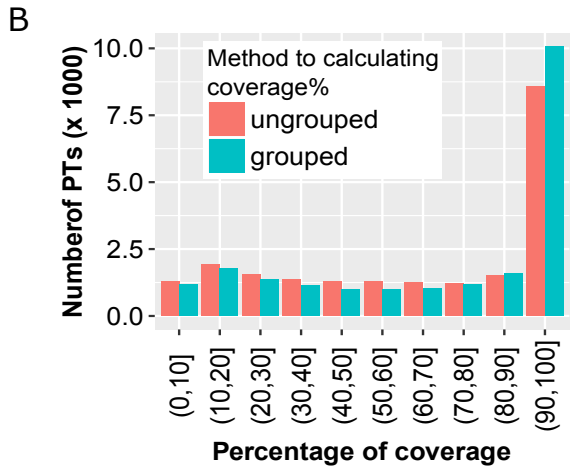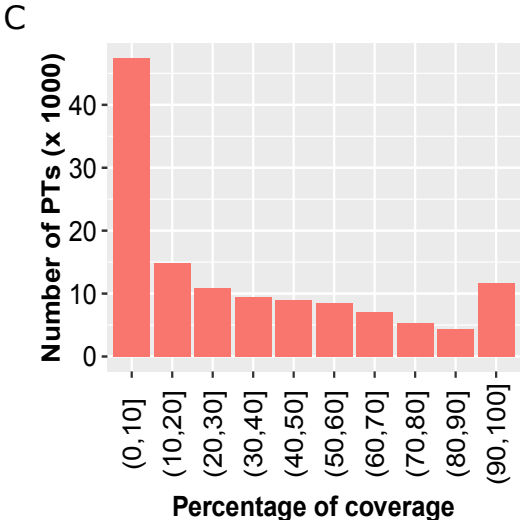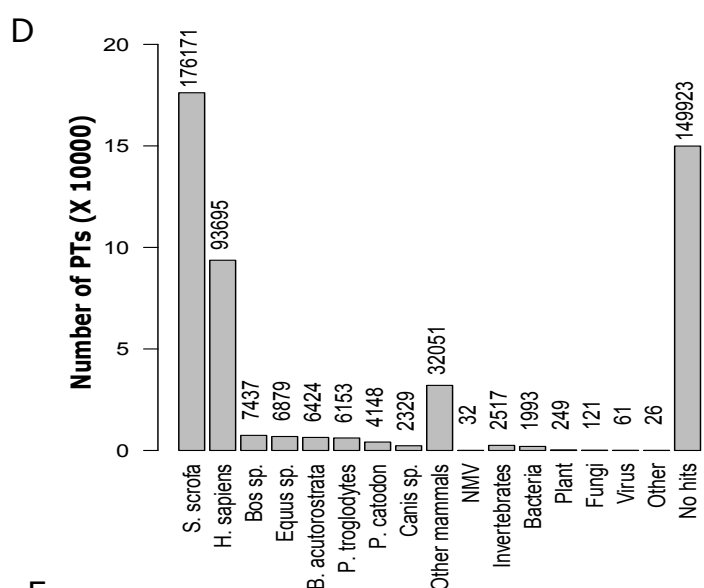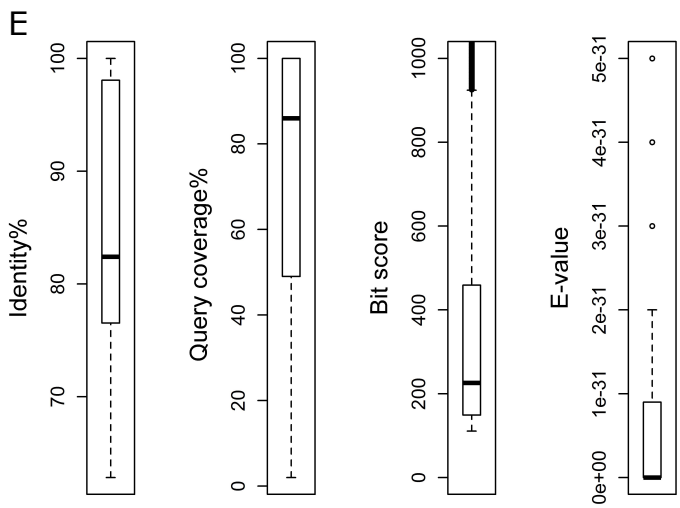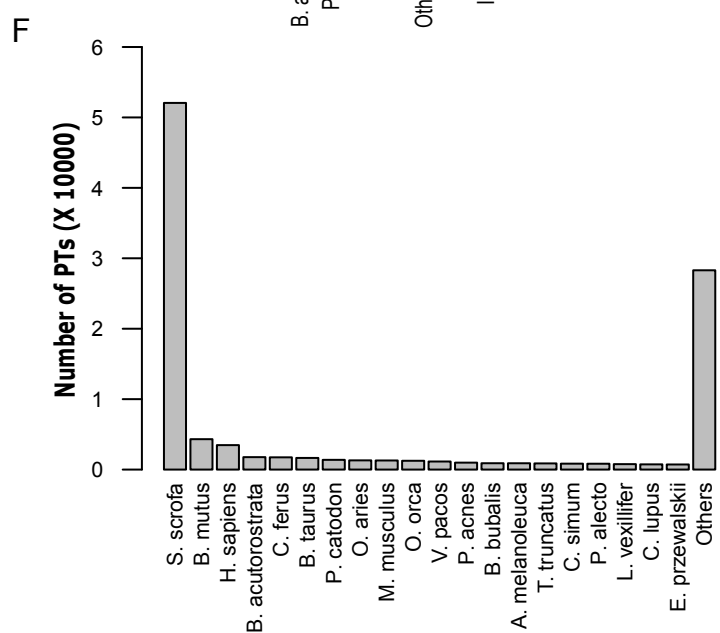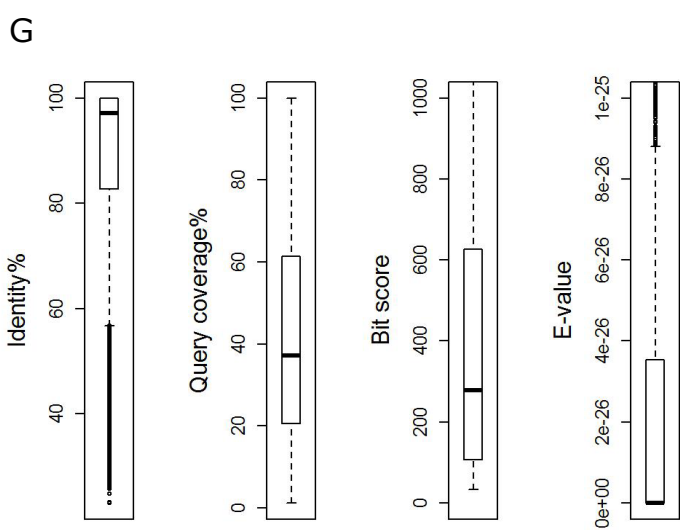

Supplement: Supplementary file 5 — Characterization of the de novo transcriptome assembly. (A) Length distribution of PTs in the de novo transcriptome assembly; (B-C) Full-length assessment by using Swiss-Prot protein (B) and pig RefSeq mRNA (C) sequences as standards. In (B), percentage of coverage of the sequences as standards by PTs were calculated based only on the best high-scoring segment pairs (HSP) (“ungrouped percentage of coverage” calculation method) or based on all HSPs (“grouped percentage of coverage” calculation method) between the two aligned sequences; (D, F) Species distribution of top DC-megaBLAST (D) and BLASTX (F) hits of the PTs in the NT and NR databases, respectively; (E, G) Boxplots showing the distributions of percentage of identity, percentage of query coverage, bit scores and E-values of the top BLAST hits of the PTs in the NCBI NT and NR databases by using DC-megaBLAST (E) and BLASTX (G), respectively. For clearer visualization, larger outliers of bit scores and E-values are not displayed. (PDF 1743 kb) [file 12864_2017_3863_MOESM5_ESM.pdf]

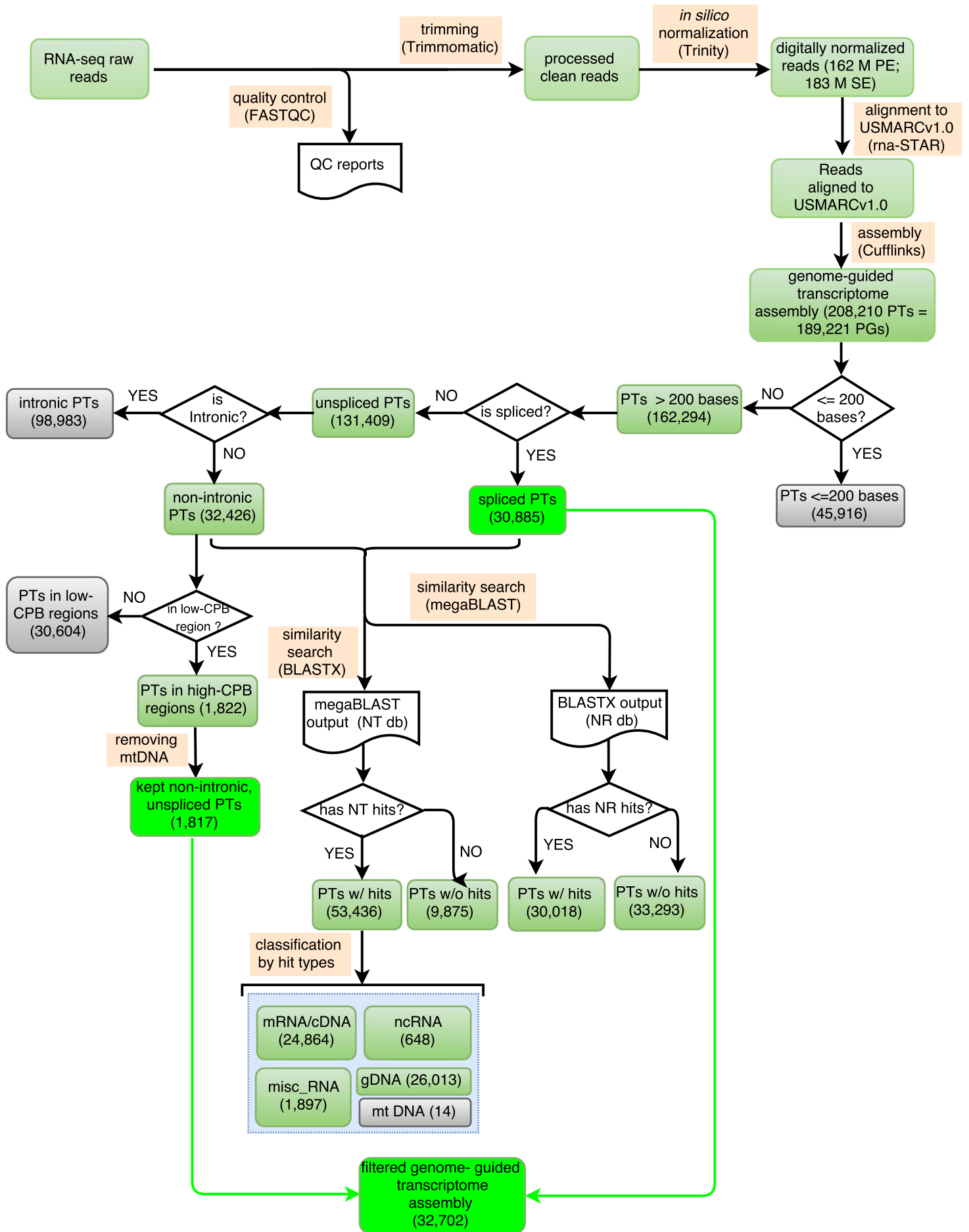

Supplement: Supplementary file 6 — Flowchart for genome-guided transcriptome assembly, annotation and filtering. The diagram shows the steps involved in construction and filtering of the genome-guided assembly, and includes the number of PTs that resulted from each step, where appropriate. Refer to the Methods section for details. The raw RNA-seq reads that were preprocessed as above (see the legend of Additional file 4: Figure S2) were mapped to the USMARCv1.0 reference genome by using STAR, and then assembled into PTs by using Cufflinks. PTs of 200 bases or shorter in length were removed from the resulting genome-guided transcriptome assembly before further analysis. Splicing status of the 162,294 PTs was determined and unspliced intronic PTs were discarded. Among the remaining PTs, those with significant BLAST hits in the NCBI NT and NR databases were determined by using DC-megaBLAST and BLASTX, with E-value cutoffs of 10−20 and 10−6, respectively. The potential biotypes of the PTs were determined based on the biotypes of their significant DC-megaBLAST hits. To complete the filtering, we removed from the remaining PTs: (i) PTs with top DC-megaBLAST hits on sequences originating from mitochondrial genomes; and (ii) PTs mapped to genomic regions of maximal CPB lower than 50× (low-CPB regions). The final filtered genome-guided transcriptome consisted of 32,702 PTs. (PDF 360 kb) [file 12864_2017_3863_MOESM6_ESM.pdf]

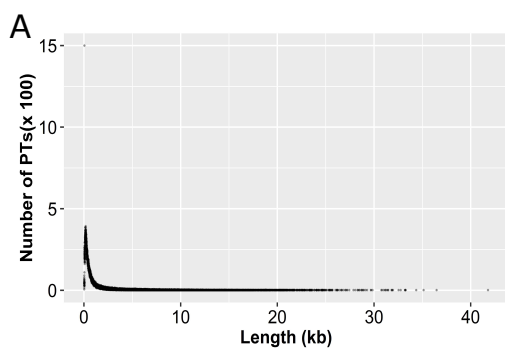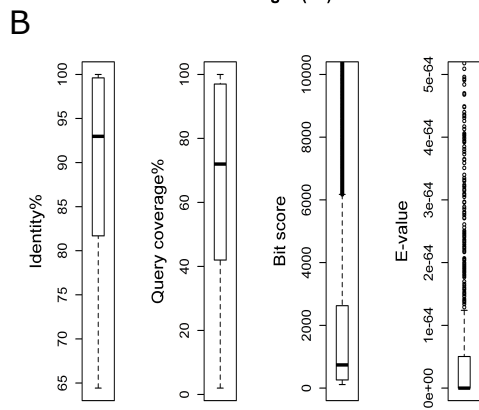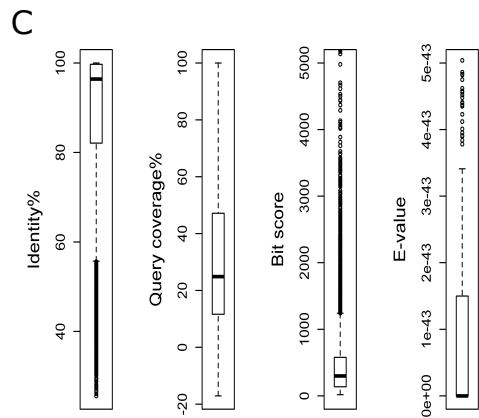

Supplement: Supplementary file 7 — Characterization of the genome-guided transcriptome assembly. (A) Length distribution of PTs in the genome-guided transcriptome assembly; (B, C) Boxplots showing the distributions of percentage of identity, percentage of query coverage, bit scores and E-values of the top BLAST hits of the PTs in the NCBI NT and NR databases by using DC-megaBLAST (B) and BLASTX (C). For clear visualization, larger outliers of bit scores and E-values are not displayed. (PDF 1877 kb) [file 12864_2017_3863_MOESM7_ESM.pdf]

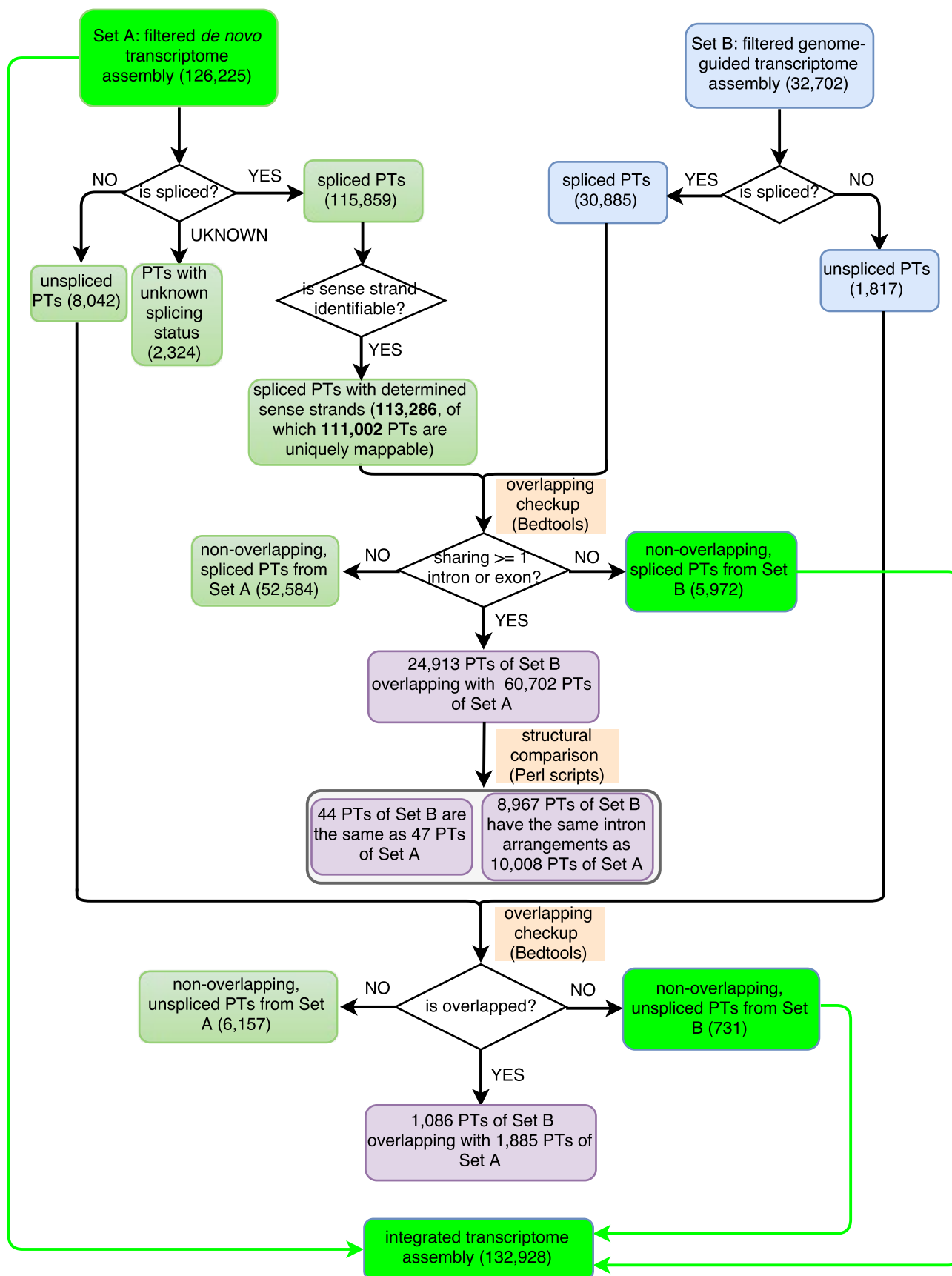

Supplement: Supplementary file 8 — Integration of the de novo and genome-guided assemblies. This diagram shows the steps used to integrate the two assemblies that were described in Figs. 1 and 2, and includes the number of PTs that resulted from each step, where appropriate; the overall goal was to identify those genome-guided assemblies that added information to the de novo assembly. The sense strands of 113,286 spliced PTs from the de novo assembly (Set A) were first determined based on consensus splice site sequences after alignment to the USMARCv1.0 reference genome. The spliced PTs in the filtered de novo transcriptome were then compared to their counterparts in the filtered genome-guided transcriptome (Set B) by using the Bedtools intersect utility and custom Perl scripts. If a spliced PT from the de novo assembly shared at least one intron or exon, or all introns and exons with a spliced PT from the genome-guided assembly mapped on the sense strand, then they were considered overlapping or exactly the same, respectively. In addition, unspliced PTs from the de novo and genome-guided assemblies were directly compared, without considering their sense strands, by using Bedtools intersect utility. PTs from the de novo transcriptome assembly, which did not overlap PTs from the genome-guided transcriptome assembly were claimed to be de novo transcriptome assembly-specific, and vice versa. The final integrated transcriptome consisted of 132,928 PTs, which included all 126,225 PTs from the filtered de novo transcriptome assembly and 6703 PTs specific to the filtered genome-guided transcriptome, including both 5972 spliced and 731 unspliced PTs. (PDF 337 kb) [file 12864_2017_3863_MOESM8_ESM.pdf]

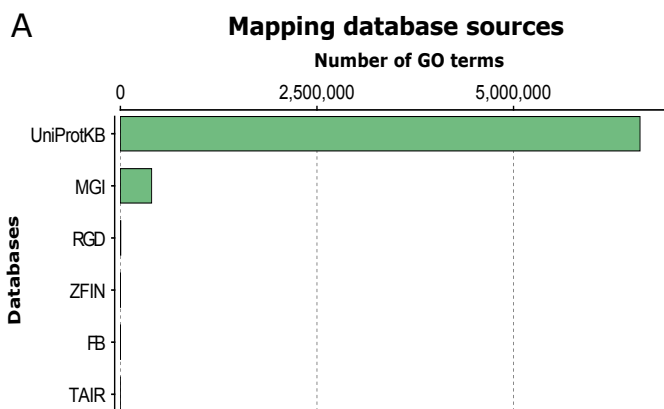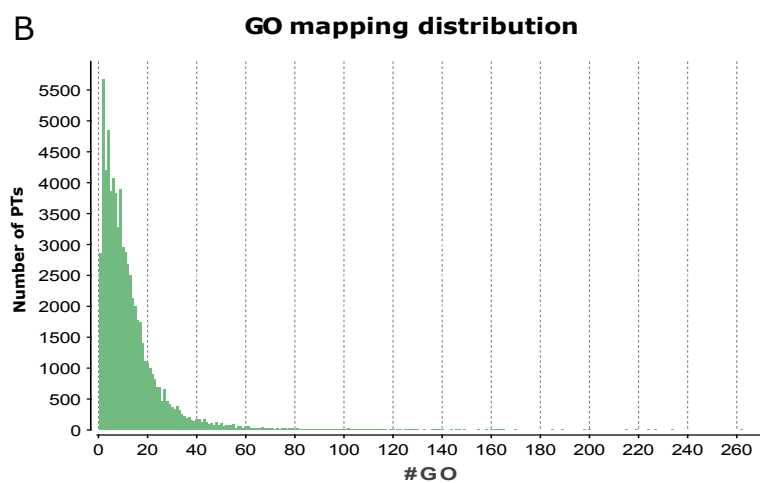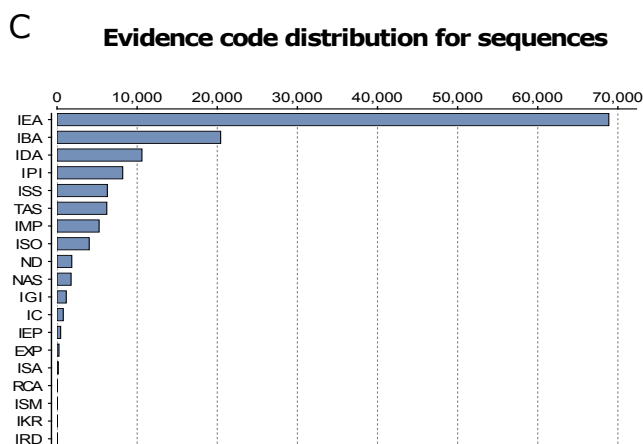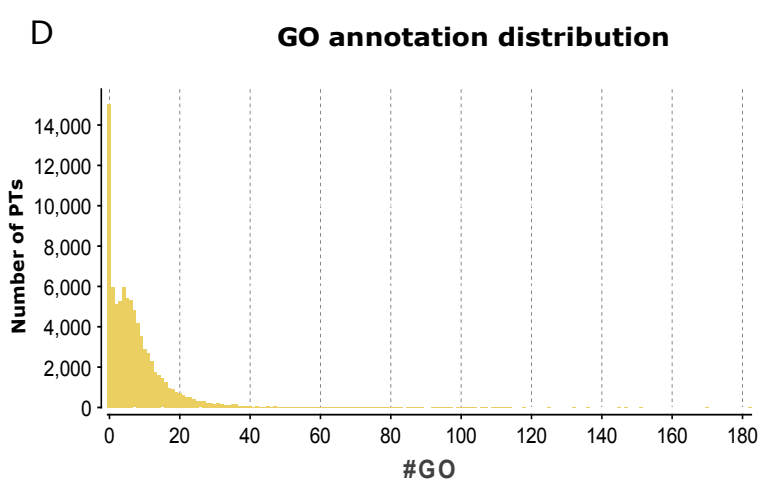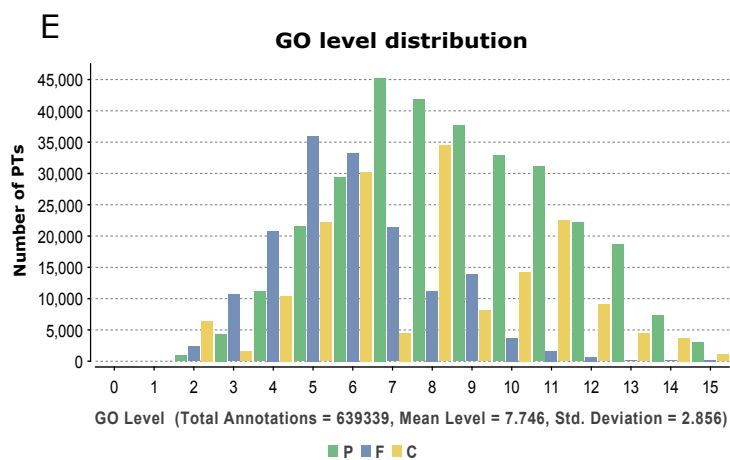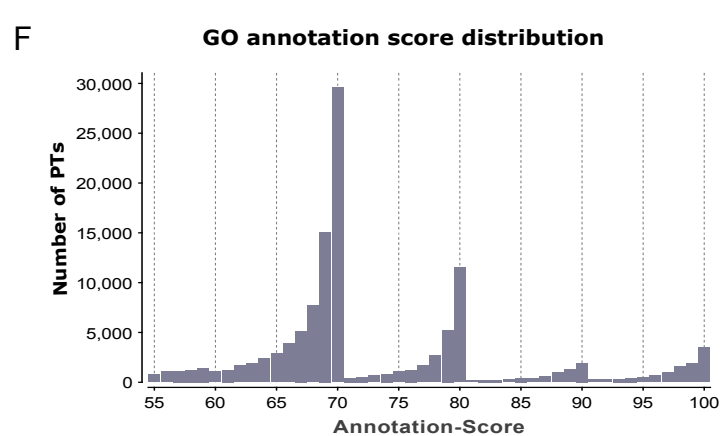

Supplement: Supplementary file 9 — Schematic summary of BLAST2GO mapping and annotation. (A) Distribution of source databases based on which GO terms were mapped to PTs in the integrated transcriptome assembly; (B) Distribution of the number of GO terms mapped to PTs of the integrated transcriptome assembly; (C) Distribution of evidence codes of the annotated GO terms mapped to PTs of the integrated transcriptome assembly; (D) Distribution of the number of GO terms mapped to PTs of the integrated transcriptome assembly; (E) Distribution of GO terms at different levels of the hierarchy of GO terms; (F) Distribution of annotation scores of GO terms. (PDF 110 kb) [file 12864_2017_3863_MOESM9_ESM.pdf]

GO-BP Terms

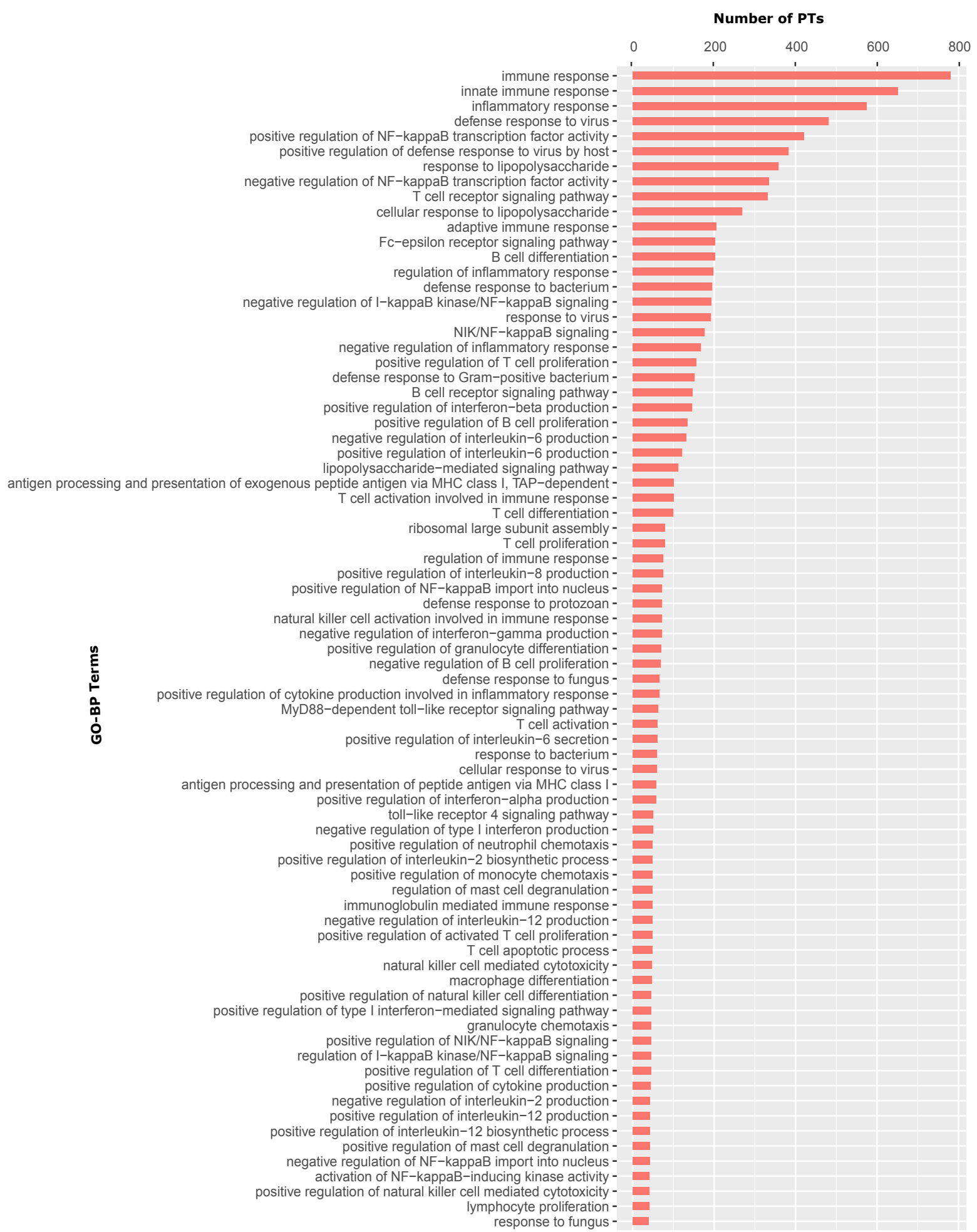

Supplement: Supplementary file 12 — Distribution of GO terms related to immune system process. (PDF 36 kb) [file 12864_2017_3863_MOESM12_ESM.pdf]

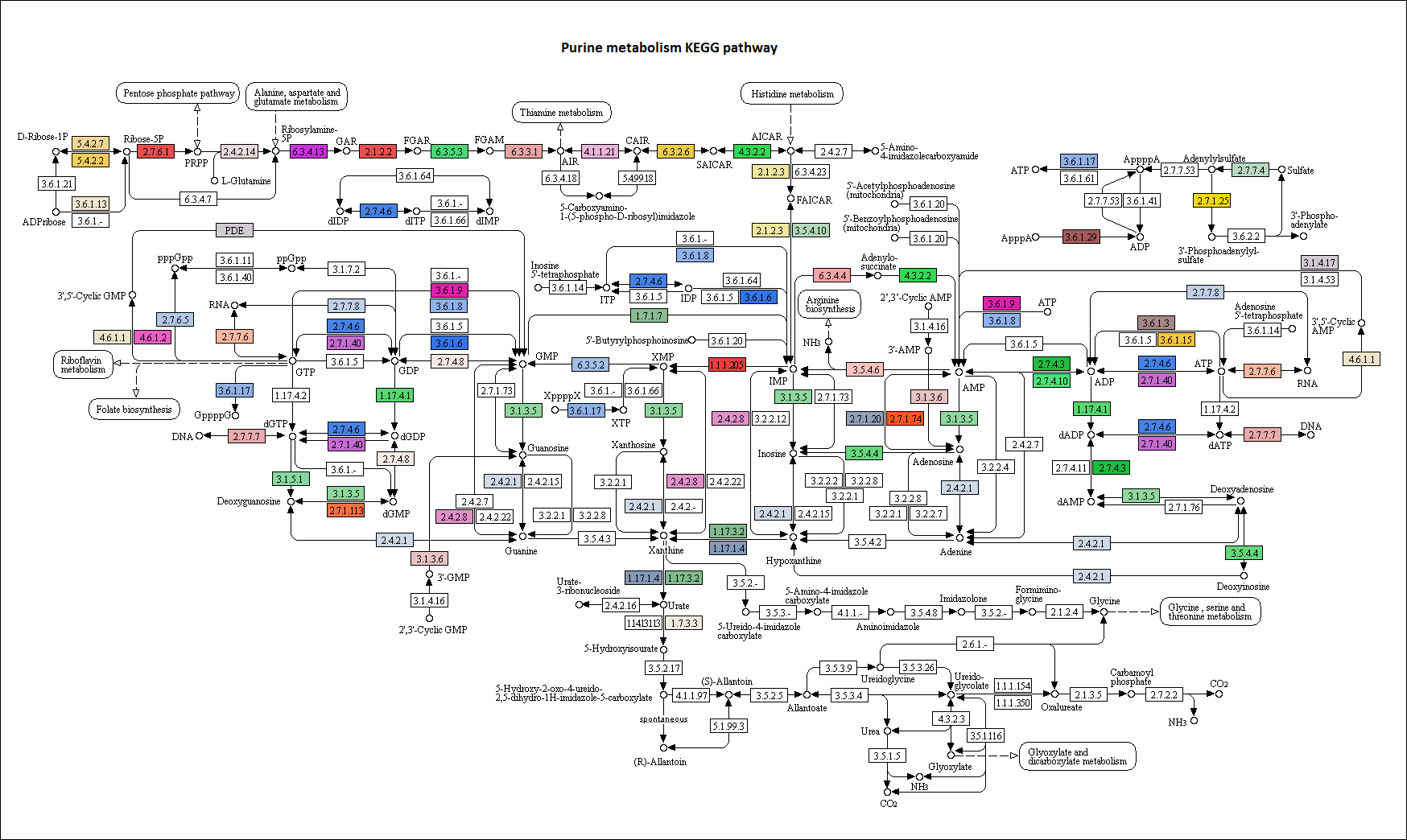

Supplement: Supplementary file 13 — The purine metabolism KEGG pathway highlighted with enzymes encoded by PTs of the integrated transcriptome. Different enzyme codes are highlighted with different colors. (PNG 88 kb) [file 12864_2017_3863_MOESM13_ESM.png]

**A**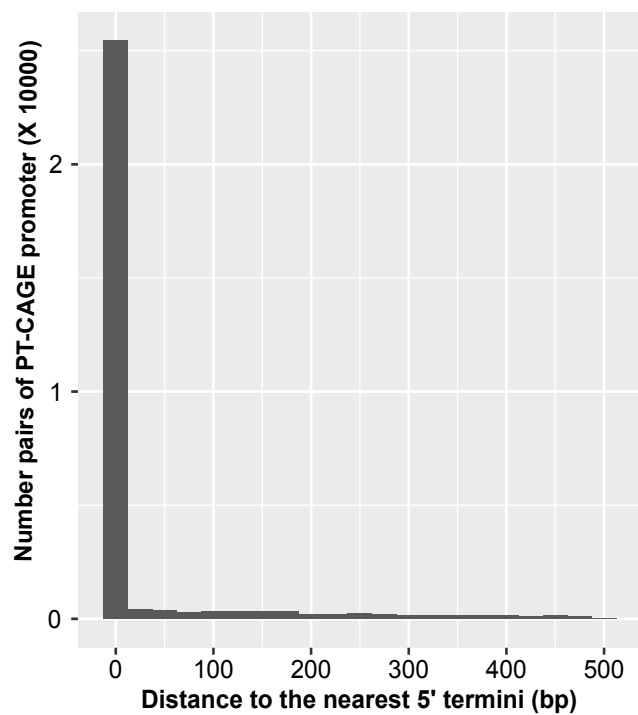**B**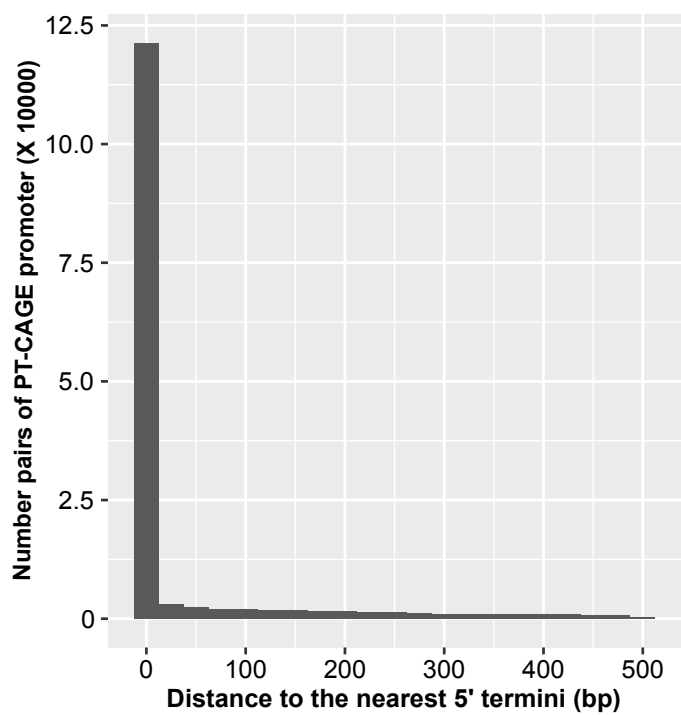

Supplement: Supplementary file 15 — The distributions of the distance between proximal promoters defined by pig macrophage CAGE data (A) or all available human/mouse/pig CAGE data (B) to the nearest 5′ termini of spliced, uniquely mapping PTs in the integrated transcriptome assembly. The vast majority of these distances were less than 50 bp, which suggested that the 5′ termini of many PTs were completely assembled. (PDF 28 kb) [file 12864_2017_3863_MOESM15_ESM.pdf]

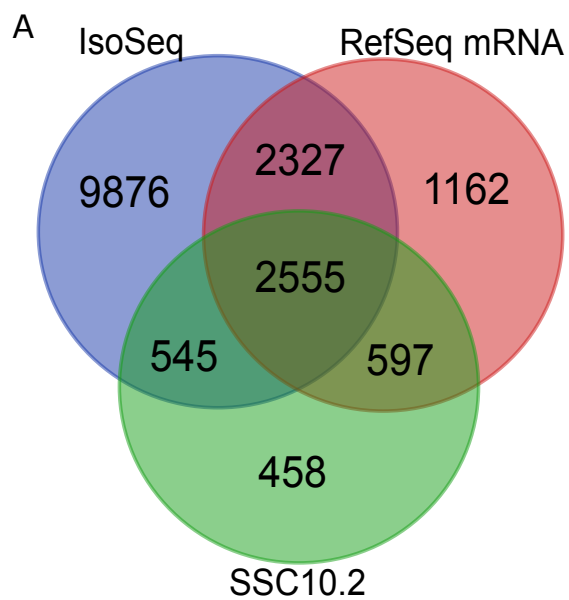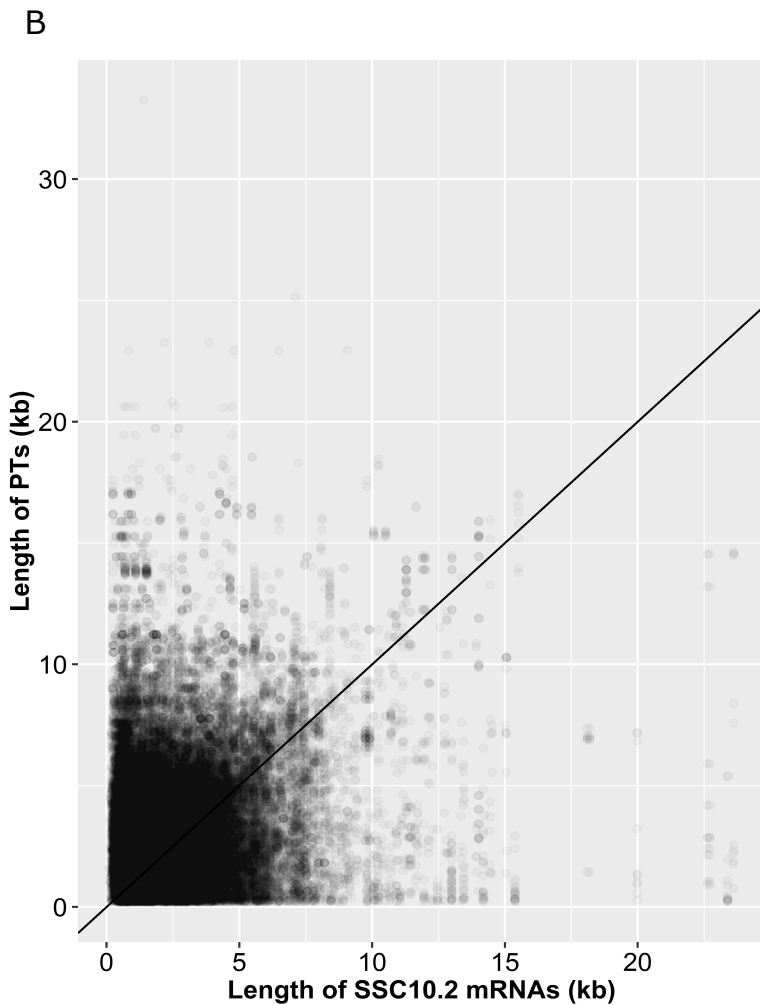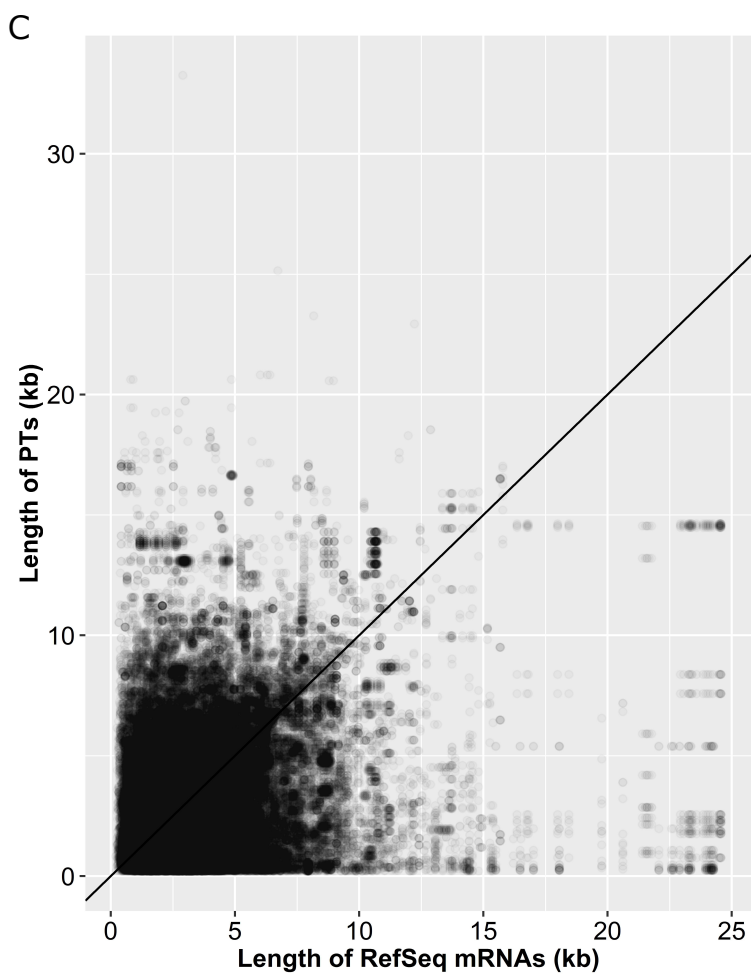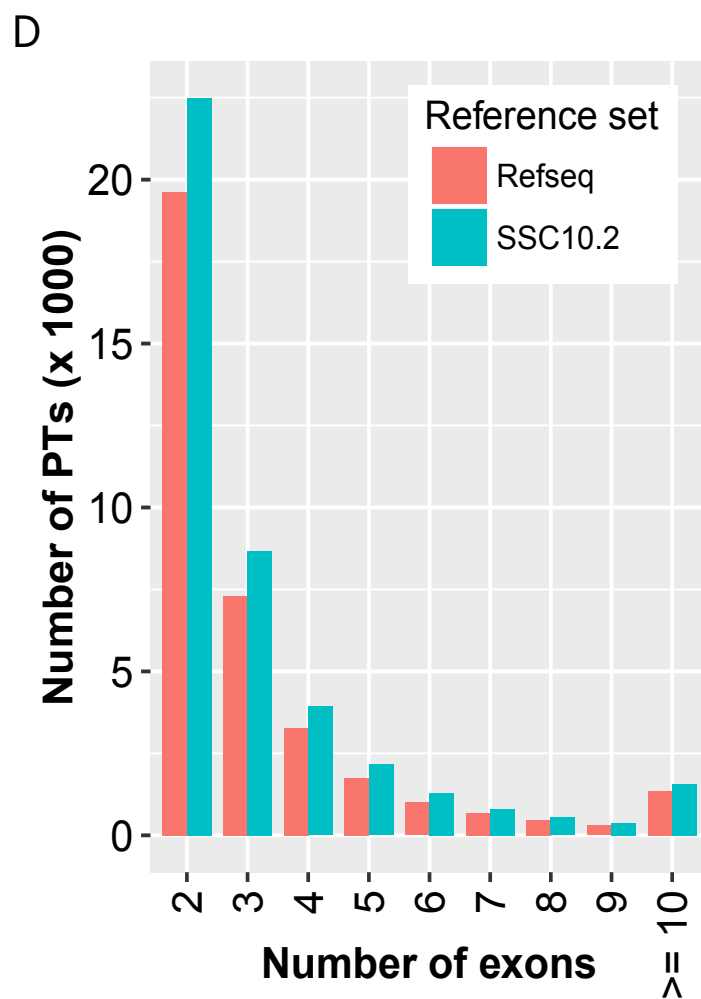

Supplement: Supplementary file 16 — Comparison of PTs of the integrated transcriptome assembly to the IsoSeq cDNA reads, transcripts annotated in Ensembl SSC10.2 and NCBI pig RefSeq mRNA sequences. (A) Venn diagram showing the number of PTs with their intron arrangements validated by the IsoSeq reads, pig RefSeq mRNA sequences and SSC10.2 transcripts; (B) Distribution of exon number of novel spliced transcripts not found in the SSC10.2 annotation or pig RefSeq mRNA set, which provides good evidence of significant extension of current genome annotation; (C) Length comparison between PTs and their maximally overlapping SSC10.2 transcripts; (D) Length comparison between PTs and their maximally overlapping RefSeq mRNA sequences. (PDF 9912 kb) [file 12864_2017_3863_MOESM16_ESM.pdf]
